# Integrative multi-omics landscape of fluoxetine action across 27 brain regions reveals global increase in energy metabolism and region-specific chromatin remodelling

**DOI:** 10.1038/s41380-022-01725-1

**Published:** 2022-09-02

**Authors:** Nirmala Arul Rayan, Vibhor Kumar, Jonathan Aow, Naghmeh Rastegar, Michelle Gek Liang Lim, Nicholas O’Toole, Edita Aliwarga, Danusa Mar Arcego, Hui Ting Grace Yeo, Jen Yi Wong, May Yin Lee, Florian Schmidt, Hajira Shreen Haja, Wai Leong Tam, Tie-Yuan Zhang, Josie Diorio, Christoph Anacker, Rene Hen, Carine Parent, Michael J Meaney, Shyam Prabhakar

**Affiliations:** 1 Genome Institute of Singapore, Agency for Science, Technology and Research (A*STAR), Singapore, 138672 Singapore; 2 Douglas Mental Health University Institute, Department of Psychiatry, McGill University, Montréal, H4H 1R3 QC Canada; 3 Department of Psychiatry, Columbia University and Research Foundation for Mental Hygiene, New York State Psychiatric Institute, 1051 Riverside Drive, New York, NY 10032 USA; 4 Singapore Institute for Clinical Sciences, A*STAR, Singapore, 117609 Singapore; 5 Brain – Body Initiative, Institute for Cell & Molecular Biology, A*STAR, Singapore, Singapore; 6 NUS Yong Loo Lin School of Medicine, National University of Singapore, Singapore, Singapore

**Keywords:** Neuroscience, Depression

## Abstract

Depression and anxiety are major global health burdens. Although SSRIs targeting the serotonergic system are prescribed over 200 million times annually, they have variable therapeutic efficacy and side effects, and mechanisms of action remain incompletely understood. Here, we comprehensively characterise the molecular landscape of gene regulatory changes associated with fluoxetine, a widely-used SSRI. We performed multimodal analysis of SSRI response in 27 mammalian brain regions using 310 bulk RNA-seq and H3K27ac ChIP-seq datasets, followed by in-depth characterisation of two hippocampal regions using single-cell RNA-seq (20 datasets). Remarkably, fluoxetine induced profound region-specific shifts in gene expression and chromatin state, including in the nucleus accumbens shell, locus coeruleus and septal areas, as well as in more well-studied regions such as the raphe and hippocampal dentate gyrus. Expression changes were strongly enriched at GWAS loci for depression and antidepressant drug response, stressing the relevance to human phenotypes. We observed differential expression at dozens of signalling receptors and pathways, many of which are previously unknown. Single-cell analysis revealed stark differences in fluoxetine response between the dorsal and ventral hippocampal dentate gyri, particularly in oligodendrocytes, mossy cells and inhibitory neurons. Across diverse brain regions, integrative omics analysis consistently suggested increased energy metabolism via oxidative phosphorylation and mitochondrial changes, which we corroborated in vitro; this may thus constitute a shared mechanism of action of fluoxetine. Similarly, we observed pervasive chromatin remodelling signatures across the brain. Our study reveals unexpected regional and cell type-specific heterogeneity in SSRI action, highlights under-studied brain regions that may play a major role in antidepressant response, and provides a rich resource of candidate cell types, genes, gene regulatory elements and pathways for mechanistic analysis and identifying new therapeutic targets for depression and anxiety.

## Introduction

Depression is a severely debilitating mental health condition that affects ~300 million individuals worldwide and is now a leading global disability burden [1, 2]. Selective serotonin reuptake inhibitors (SSRIs) such as fluoxetine (FT) are routinely prescribed for depression, as well as for a range of co-morbid conditions such as anxiety and bipolar disorder [3, 4]. Approximately 81% of patients diagnosed as depressed receive at least one prescription for antidepressants (ADs), with SSRIs constituting 60% of such prescriptions (~250 million people worldwide) [5, 6]. Moreover, SSRIs have pronounced side effects, including mental sluggishness, sexual dysfunction and increased suicidality, perhaps indicating that they have complex effects on multiple brain regions [7, 8]. It is thus important to develop novel drugs and drug combinations that could deliver the beneficial effects of SSRIs with lower rates of treatment failure and fewer side effects [9].

A major hurdle in the development of alternative therapeutics is that the mechanism of action of SSRIs is not well characterised [9–12]. For example, although their clinical benefit was initially attributed to inhibition of serotonin reuptake [13–15], multiple additional mechanisms of action have subsequently been proposed, including enhanced adult neurogenesis and increased synaptic plasticity [16–20]. Even this list of candidate mechanisms is almost certainly incomplete, for reasons described below. It is thus imperative that a comprehensive, unbiased analysis of the molecular landscape of SSRI effects across the brain is performed, to advance our understanding of the biology of SSRI response and support the development of new therapeutics.

In agreement with the diversity of proposed mechanisms, multiple studies have shown that commonly-used antidepressants can alter the expression of few hundreds of genes [21–23], potentially by inducing epigenetic alterations [24, 25]. However, one major limitation is that previous studies of SSRI action have focused on a limited set of candidate brain regions or a limited set of gene loci [22, 26, 27]. Moreover, omics analyses of SSRI action are exclusively unimodal, i.e. based either on gene expression or epigenetic profiling, but not both [23, 26, 27]. Lastly, these omics studies rely exclusively on bulk-tissue profiling, which limits our ability to identify the underlying alterations in cell type abundance and cell-type-specific gene regulatory networks. Nevertheless, there is evidence that antidepressants induce a substantial number of molecular alterations in multiple brain regions, including changes in chromatin state and gene expression [28, 29]. Thus, a comprehensive, multimodal characterisation of gene regulatory changes associated with SSRI treatment, integrating both bulk and single-cell approaches, could reveal avenues for identifying novel targetable pathways and molecules [30–32]. The use of naïve, healthy animals in such an approach limits common confounds known to be associated with current models of depression [33].

We report a comprehensive multi-omics map of the molecular effects of fluoxetine on rat brain, a widely-used model of human depression and antidepressant response [34–36]. We profiled gene expression (bulk RNA-seq, 210 datasets) and chromatin state (bulk chromatin immunoprecipitation sequencing (ChIP-seq) for the histone marker H3K27ac, 100 datasets) in a broad, unbiased panel of 27 brain regions across the entire rodent brain, in naive and fluoxetine-treated animals. We complemented this approach with single-cell RNA-seq (scRNA-seq) analysis of two of the major zones of neuronal proliferation in the adult brain: the dorsal and ventral dentate gyri of the hippocampus [37]. Using diverse integrative data analysis techniques and comparisons to human genome-wide association studies (GWAS) and the Psychiatric disorders and Genes association NETwork (PsyGeNET), we characterised the complex and multifaceted effects of fluoxetine on region-specific and cell-type-specific gene regulatory networks and pathways. Remarkably, we observed profound molecular changes across the brain (>4000 differentially expressed genes and differentially acetylated ChIP-seq peaks each) that were highly region-dependent, with the raphe, nucleus accumbens, locus coeruleus and dorsal hippocampus emerging as the most strongly altered by fluoxetine. We observed a global shift in pathways related to histone and chromatin modifications, metabolism, and mitochondria, suggesting chromatin remodelling and increased energy production in 24/27 brain regions upon administration of fluoxetine. In bulk and single-cell analyses, specific oligodendrocyte and neuronal subtypes emerged as the major responders to fluoxetine. We also detected a steep gradient in molecular responses to fluoxetine along the dorso-ventral axis of the hippocampus. These results provide the first comprehensive map of the molecular effects of fluoxetine on the mammalian brain and suggest new directions for mechanistic investigation and eventual therapeutics development.

## Methods

### Animal housing and treatment

All procedures were performed in accordance with the guidelines established by the Canadian Council on Animal Care with protocols approved by the McGill University Facility Animal Care Committee. Long-Evans rats were purchased from Charles River (RRID:RGD_2308852) and bred at the Douglas Mental Health University Institute animal facility. 60-day old uncharacterised male rats were housed in pairs and separated as control (Sham) group and fluoxetine-treated (FT) group. Fluoxetine (18 mg/kg/day) was provided ad libitum in the drinking water and this formed the treatment group, while rats in the Sham group received only water. See Supplementary Methods for details on materials, animal handling and behavioural assessments.

### Bulk RNA-seq

Frozen, pooled brain tissue punches for each region were processed for RNA extraction and subsequent cDNA synthesis (Supplementary Methods, *n* = 4 replicates pooled from 40 animals per treatment group). cDNA libraries were prepared using 300 ng of total RNA, from 27 regions in every replicate. Multiplexed RNA-seq libraries were sequenced as paired-end, 76 bp reads on Illumina HiSeq 2500 v4. QC and all downstream data analysis pipelines are detailed in Supplementary Methods.

### Bulk ChIP-seq

For each ChIP-seq assay ~5–25 mg of frozen brain tissue per replicate per region was processed for pulldown assay (*n* = 2 replicates pooled from 20 animals per treatment group). ChIP and subsequent library preparation was performed as described in Supplementary Methods as well as here [38]. Protein-DNA complexes were immuno-precipitated using 3 µg of H3K27ac antibody of the same lot no. for all 108 (27 regions × 2 replicates × 2 treatment groups) ChIP experiments. Multiplexed ChIP-seq libraries were sequenced as paired-end, 76 bp reads on Illumina HiSeq 2500 v4. QC and all downstream data analysis pipelines are detailed in the Supplementary Methods.

### Single cell RNA-seq

dorDG and venDG tissues were punched from fresh brains of the two treatment groups (*n* = 5 replicates pooled from 15 animals per treatment group). Single cells gel emulsions and their cDNA were isolated following manufacturer’s recommendations for the 10x Genomics Chromium single cell 3’ reagent kit v2. Single cell libraries were sequenced on the Illumina HiSeq 4000. QC and all downstream data analysis pipelines are presented in the Supplementary Methods.

## Results

### Transcriptomic landscape of fluoxetine action across 27 brain regions

#### Affected brain regions and magnitude of region-specific changes

**Fig. 1:**
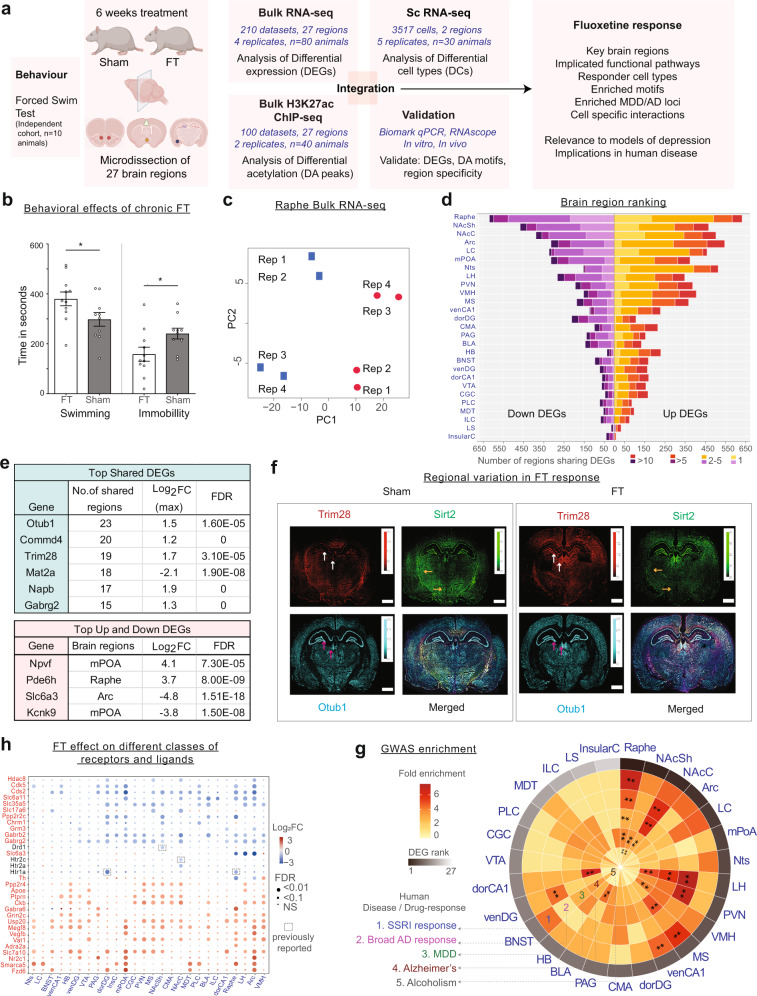
Genome-wide transcriptome changes by fluoxetine. **a** Schematic overview of data generation and workflow. **b** Forced swim test following chronic fluoxetine treatment in adult rats. Time spent swimming, being immobile are shown for sham and fluoxetine treated groups. * indicates *P-val* < *0.05*. **c** RNA-seq dataset QC: Principal Component Analysis (PCA) visualisation of eight samples of the raphe region. Sham replicates are coloured in blue and FT coloured in red. **d** Statistics of RNA-seq dataset: Number of upregulated and downregulated DEGs across 27 brain regions (and number in each category shared between regions). See Supplementary Tables TS1a for brain region names. **e** *Top:* table showing DEGs shared in ≥15 regions, along with the maximum log_2_FC and its corresponding FDR. *Bottom:* table showing strongest upregulated and downregulated region-specific DEGs. **f** Regional variation in fluoxetine DEGs as measured by RNAscope. For each gene, arrows denote regions with significant differential expression between FT and Sham that are consistent with bulk RNA-seq results. **g** Circular heat map showing the fold-enrichment of identified region-wise DEGs in GWAS loci for five phenotypes. * indicates FDR *Q-val* ≤ 0.05; ** FDR *Q-val* ≤ 0.01. **h** Differential expression status of key neurotransmission genes identified. Grey boxes denote previous findings reported in the literature (see Supplementary Tables TS6).

To confirm the behavioural effects of fluoxetine, we treated naïve rats with either vehicle or fluoxetine for 6 weeks, and tested behavioural despair using the forced-swim test (Fig. 1a) [39]. Animals that received chronic fluoxetine treatment for 6 weeks showed a significant increase in swim time and a corresponding reduction in immobility compared to control animals (*P-val* < 0.05, Fig. 1b, Supplementary Table TS1). Thus, our fluoxetine treatment of naïve animals successfully reproduced the well-known reduction in behavioural despair induced by antidepressants [39].

Next, to comprehensively characterise the genome-wide transcriptomic response to fluoxetine, we used bulk RNA-seq to profile four treated (FT) and four control (Sham) samples from each of 27 regions spanning the entire rat brain (27*8 = 216 transcriptomes; Fig. 1a, Supplementary Table TS1). To reduce the effects of inter-animal biological variation within treatment groups, each sample was pooled from ten animals (40 in Sham, 40 in FT; 80 animals in total). Most datasets were found to be of high quality; only six RNA-seq profiles were discarded during quality-control (QC, Supplementary Methods). As expected, the averaged transcriptomes of the 27 brain regions formed distinct clusters based on anatomical proximity (Supplementary Fig. S1a). For example, cortical regions clustered with the striatal and hippocampal substructures and were relatively distant from the thalamic and caudal nuclei. Thus, the data were consistent with the expected anatomical relationships in the brain.

Within each brain region, the transcriptomes of treated and untreated samples were clearly distinct, indicating strong and widespread gene expression changes in response to fluoxetine (Fig. 1c, d, Supplementary Fig. S1b, Supplementary Table TS2). Strikingly, as many as 4447 transcripts were altered by antidepressant treatment in at least one brain region (absolute log_2_ fold-change (log_2_FC) ≥ log_2_ (1.25), false discovery rate (FDR) *Q-val* ≤ 0.1). The median number of differentially expressed genes (DEGs) in any individual brain region was 311 (Fig. 1d, Supplementary Table TS2). These statistics imply that fluoxetine has strong effects on gene expression that vary substantially across brain regions.

The brain region where the transcriptome was most strongly affected by fluoxetine treatment was the raphe (1243 DEGs, Fig. 1d, Supplementary Table TS3). The next four brain regions, in descending order, were nucleus accumbens shell (NAcSh) and core (NAcC), arcuate nucleus (Arc) and the locus coeruleus (LC). Notably, all of the five brain regions highlighted by this unbiased molecular analysis are major monoaminergic centres [40, 41]. Thus, our transcriptomic analysis supports the centrality of monoaminergic signalling to fluoxetine response, including brain regions such as LC [42] and Arc [43] that have not previously been characterised in omics studies of fluoxetine response (Fig. 1d).

We hypothesised that some DEGs may be shared across brain regions, due to sharing of cell subtypes and long-range neural circuitry. Indeed, we found that, while 47% of DEGs were specific to a single brain region, 33% were altered in three or more regions (Fig. 1e, Supplementary Table TS4a). Intriguingly, *Otub1*, the most broadly upregulated gene (23/27 regions), contributes to neurogenesis and elevates protein synthesis and cellular metabolism [44, 45]. *Trim28* (*KAP1*), an epigenetic co-repressor gene upregulated in 19 brain regions, has a knockout phenotype of heightened anxiety [46]. Thus, two of the three most broadly upregulated DEGs are functionally associated with hallmark therapeutic effects of fluoxetine treatment. Remarkably, amongst the most broadly downregulated genes, four of the top five annotated transcripts contribute to neurotransmitter synthesis, packaging or release: *Mat2a* [47], *Napb* [48], *Cds2* [49] and *Gabrg2* [50] (Fig. 1e). Their downregulation could potentially dampen serotonin release at synapses as part of a homoeostatic negative feedback loop [51, 52] compensating for the perturbation introduced by drug treatment.

Fluoxetine-responsive genes with the strongest region-specific upregulation were *Npvf* (log_2_FC:4.1, mPoA), a neuropeptide gene in the hypothalamic circuit regulating pain, sleep and appetite [53], and *Pde6h* (log_2_FC:3.7, raphe), a phosphodiesterase gene that regulates light sensitivity and pain perception [54]. *Slc6a3* (log_2_FC:-4.8, Arc), the dopamine reuptake transporter gene [55], and *Kcnk9* (log_2_FC:-3.8, mPoA), a potassium channel gene implicated in sleep regulation [56], showed the strongest region-specific downregulation in response to fluoxetine. Thus, in addition to monoamine signalling, the most strongly altered genes are involved in pain perception and sleep regulation, two well-known targets of fluoxetine [7]. Indeed, SSRIs are prescribed for problems of chronic pain and to enhance sleep quality [57, 58]. Overall, the above results reveal considerable variation in the molecular response to fluoxetine across brain regions and provide novel mechanistic hypotheses for the known therapeutic and adverse effects of fluoxetine (Fig. 1e, Supplementary Table TS4b, and Supplementary Fig. S2).

To assess the reliability of our expression analysis, we prioritised 96 top-ranked DEGs from six brain regions (8 up and 8 downregulated genes per region, Supplementary Methods) for independent validation using the Fluidigm Biomark qPCR assay. We quantified the expression of the 96 genes in the corresponding brain regions and observed that expression fold-changes in the qPCR assay were consistent with “Up” and “Down” status in the RNA-seq dataset (Supplementary Fig. S3a). To further corroborate our results, we performed single-molecule RNA-FISH (RNAscope) on brain sections from sham and fluoxetine-treated animals. For this analysis, we prioritised the abovementioned *Otub1* and *Trim28* genes, as well as *Sirt2*, a DEG that may play a role in depressive behaviour and modulation of chromatin by SSRIs [59]. All three genes showed substantial region-specific changes in expression consistent with their respective bulk RNA-seq status (Fig. 1f, Supplementary Fig. S3b–e). Thus, these independent assays support the robustness of our DEG set.

#### Correspondence with independent analyses

To assess relevance to human clinical phenotypes, we asked whether the DEGs identified in our data overlapped with those identified by GWAS. We examined GWAS gene sets and summary statistics for five phenotypes: SSRI response, broad antidepressant response, MDD, Alzheimer’s and alcoholism (Supplementary Methods). In seven brain regions, we found highly significant overlap (FDR *Q-val* ≤ 5e-5) between our DEGs and those associated with SSRI/antidepressant response. Notably, six of these seven regions ranked above the median in terms of number of DEGs, including two monoaminergic-related centres (raphe and NAcC, Fig. 1g). The identification of the raphe as a highly-enriched region is in agreement with reports of SSRI effects on Htr1a autoreceptor regulation in this region [60]. Consistent with the idea that antidepressants target molecular processes dysregulated in depression [61], DEGs in four of the above six regions (raphe, NAcC, LH and MS) also showed highly significant overlap with MDD genes. Intriguingly, DEGs in three of the four brain regions were enriched for genes associated with Alzheimer’s. This result is potentially attributable to the genetic relationship between Alzheimer’s and depression [62] and consistent with the use of fluoxetine as a treatment for Alzheimer’s [63]. Finally, two regions (raphe and NAcC) showed significant overlap between DEGs and genes associated with alcoholism, which is often co-morbid with MDD [64] (Fig. 1g, Supplementary Table TS5). In addition, to capture overlap with neuropsychiatry gene sets beyond GWAS, we performed enrichment analyses using the PsyGeNET database (Supplementary Methods). We observed a significant enrichment between our DEGs and curated gene sets linked to depressive phenotypes, bipolar disorder, schizophrenia and cocaine (min FDR *Q-val*: 0.0027, Supplementary Table TS5, Supplementary Fig. S5a). Interestingly, some of the overlapping genes are known targets of drugs used in the treatment of psychiatric disorders (*see Discussion*). Overall, these results indicate strong correspondence between our DEG sets and genes related to human depression, treatment response and therapeutic relevance in psychiatric disorders.

Next, we examined the overlap in gene expression changes between our data from naïve rats and antidepressant-response studies of stressed mice [65, 66] (Supplementary Fig. S6, Supplementary Methods). Transcriptional alterations associated with effective behavioural response to fluoxetine in stressed mice (FT responders in stressed mice (corticosterone +FT); [66]) showed a significant overlap with the naïve fluoxetine response, with a stronger concordance (*max(–log*_*10*_*p-value*) = 18.3) among the downregulated genes in the hippocampus (Supplementary Fig. S6a). This finding suggests similarity in SSRIs response across both naïve and stressed animals. Next, to assess the influence of a different stress model and non-SSRI treatment, we performed the overlap analysis with gene expression changes associated with responders to the tricyclic antidepressant, imipramine [65]. We again observed a strong concordance in gene expression changes in 6/7 brain regions examined, particularly in the amygdala (BLA, CMA; *max (–log*_*10*_*p-value)* *=* *52.6*) and the nucleus accumbens (NAcC, NAcSh; *max (–log*_*10*_*p-value)* *=* *54.6*) (Supplementary Fig. S6b) suggesting common gene regulatory mechanisms between fluoxetine and imipramine. Finally, we compared differential expression in our data with fluoxetine-induced changes in mouse venDG following chronic variable stress (Anacker et al., unpublished). We once again observed significant overlap within both up-and down-regulated genes (*max (–log*_*10*_*p-value)* *=* *12.4*) across datasets (Supplementary Fig. S6c). Taken together, the analyses suggest that the gene expression changes in fluoxetine-treated naïve rats overlap substantially with antidepressant treatment in several paradigms of stressed rodents.

Lastly, we examined the correspondence between previously reported effects of SSRIs on serotonin receptors and our datasets. In the hippocampus and raphe, fluoxetine and other SSRIs are known to decrease *Htr1a* receptor expression and serotonin binding. Moreover, Htr1a blockade reverses depressive behaviours in mice [60, 67, 68]. Consistent with this, we observed a significant reduction in *Htr1a* expression in the raphe and dorDG (Fig. 1h). Similarly, we recapitulated the previously reported SSRI-induced downregulation of *Htr2c* in NAcC [69–71] (Fig. 1h). Examining a broader set of signalling receptors, we find that fluoxetine significantly modulates receptor expression in a plethora of brain regions not previously subjected to unbiased transcriptome profiling. These include broad downregulation of the GABA receptor subunits *Gabrb2* and *Gabrg2* in >10 regions and upregulation of the NMDA receptor subunit *Grin2c* in 7 regions, as well as *Grm3* and *Chrm*1 in 3 regions (Fig. 1h, Supplementary Fig. S4a). We also detected numerous region-specific alterations in the expression of Wnt-Notch (*Fzd6*, *Megf8*, *Lrp5*), purinergic (*Adora2a*, *P2ry1*), nuclear hormone (*Nr2c1*, *Nr4a2*) and transmembrane protein kinase (*Bmpr1a, Ntrk2*) signalling receptors, as well as their downstream solute carriers (*Slc6a11*, *Slc7a10*) and metabolite regulators (*Apoe*, *Cds2*) (Fig. 1h, Supplementary Fig. S4b, Supplementary Table TS6). These results demonstrate the diversity of fluoxetine’s effects and highlight novel fluoxetine-responsive receptor categories for further investigation.

#### Cell type and pathway signatures of fluoxetine response

We asked if fluoxetine-induced DEGs in the 27 brain regions could be matched to specific cell types. To address this, we tested for enrichment of DEGs in cell-type markers derived from the BRETIGEA database [72] (Supplementary Methods). In multiple brain regions, upregulated DEGs were significantly enriched for oligodendrocyte (6 regions), neuronal (5 regions) and microglial (3 regions) markers. Downregulated DEGs were overwhelmingly enriched for neuronal (17 regions) and oligodendrocyte (12 regions) markers (Supplementary Fig. S5b, Supplementary Table TS7). Together, these results extend previous low-throughput studies that suggested oligodendrocyte and neuronal alterations in depression and antidepressant response [73]

MDD and rodent stress models report dysregulation of oligodendrocyte-specific genes, including those related to myelin, in the NAc as a whole [74, 75]. However, the NAcC and NAcSh are functionally distinct subregions with disparate roles in regulation of depression and addiction [76, 77]. Indeed, we observed distinct fluoxetine responses in these subregions. Upregulated DEGs in NAcC were strongly associated with oligodendrocytes (FDR *Q-val: 1.5e-20*), whereas those in NAcSh were enriched for neuronal markers (FDR *Q-val: 6.6e-6*) (Supplementary Fig. S5b, Supplementary Table TS7). Thus, our results suggest that fluoxetine may influence distinct cell types in these two components of the NAc.

**Fig. 2:**
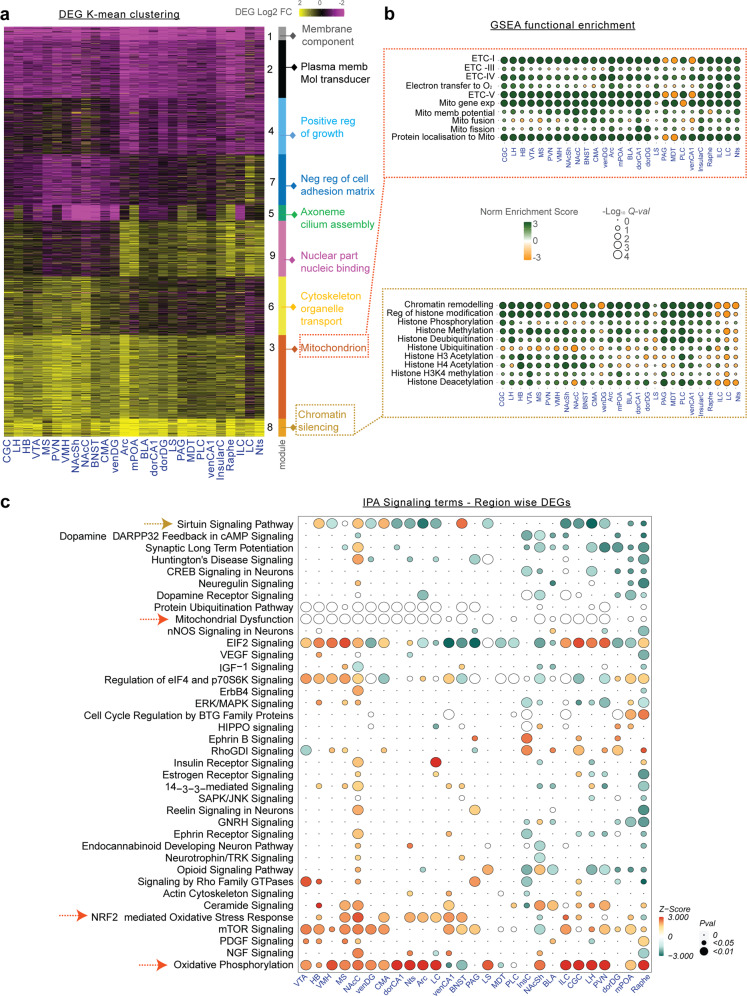
Functional signatures of fluoxetine induced transcriptome alterations. **a** k-means clustering of the union set of region-wise DEGs. Top gene ontology terms are shown for each co-regulated gene module. **b** GSEA enrichment ratios of upregulated (*green bubble*) and downregulated (*yellow bubble*) DEG sets with their corresponding functional activity (normalised enrichment score, NES) per the GO database. Circle size indicated –Log_10_(*Q-val*) and the colour indicates NES. **c** Pathway enrichment analysis of region-wise DEGs using IPA. Pathways that had at least five genes in the foreground DEG set and with FDR *Q-val* < 0.01 in >3 regions are represented in the plot. Green circles denote positive z-score (pathway activation), red circles denote negative z-score (pathway inhibition) and white denotes neutral score. Red arrows indicate broadly altered pathways related to energy metabolism, while tan arrow indicates pathways related to chromatin modifications.

Next, we asked if fluoxetine-induced DEGs could elucidate molecular pathways modulated by the drug. To determine the global relationship between DEGs, we used k-means clustering of all 4,447 DEGs to identify co-regulated gene modules (Fig. 2a). For each module, we detected enriched functional categories using anRichment (FDR *Q-val* ≤ 0.05, Supplementary Methods) and GOrilla (FDR *Q-val* ≤ 0.05, enrichment ratio ≥ 1.5, Supplementary Methods). We used gene set enrichment analysis (GSEA, Supplementary Methods) on GO categories functionally related to the k-mean modules (Fig. 2b). Finally, we complemented the above module-centric analyses by using Ingenuity Pathway Analysis (IPA) to identify pathways enriched in region-wise DEGs (Fig. 2c, Supplementary Table TS9, Supplementary Methods).

Strikingly, we observed two DEG modules (module 3 and 8) upregulated in almost all 27-brain-regions (Fig. 2a). Module 3 was enriched for genes with mitochondrial functions (GO:0005739, FDR *Q-val:1.4e-6*) such as electron transport complex (ETC) subunits (*Ndufb4, Ndufa7, Uqcrq, Atp5f1, Sdhb*), inner and outer mitochondrial membrane genes (*Tomm6, Timm17a*), mitochondrial matrix components (*Mdh2, Mrp15, Mrps7*) and transport chaperones (*Sirt1, Dnajc3, Dnajc19*) (Fig. 2a, Supplementary Table TS8a, c). Correspondingly, GSEA analysis showed enrichment for gene sets modulating ETC I-V activity and mitochondrial protein import (Fig. 2b, Supplementary Table TS8b, c). In parallel, IPA highlighted pathways related to mitochondrial function as systematically altered in the majority of brain regions (red arrows, Fig. 2c). In particular, the oxidative phosphorylation (oxphos) pathway was significantly upregulated in 19/27 regions, suggesting a widespread increase in energy production upon fluoxetine administration. Antidepressant-induced higher energy levels positively regulate mTOR signalling [78]. Consistent with this, we observed an upregulation of mTOR signalling across >15 regions. Increased energy production often leads to cellular damage from oxidative stress. Accordingly, IPA inferred a significant increase in oxidative stress response across brain regions (Fig. 2c, Supplementary Table TS9, *P-value* ≤ 3.6e-4 in 12/27 regions). Thus, gene-module and region-wise DEG analysis consistently suggest a near-global increase in energy production and related antioxidant defences as one of the most prominent molecular effects of fluoxetine administration. Importantly, these effects seem primed to oppose the downregulation of oxphos genes previously reported in MDD (*Atp5j2, Atp5i, Ndufb4, Ndufa5, Mt-atp8*) and the well-known depletion of ATP in MDD brain [79, 80].

The second co-regulated gene cluster that exhibited global upregulation across brain regions was module 8 (Fig. 2a). 14/195 DEGs within this module are transcription factors (*MSigDB*). GSEA showed that upregulated genes were significantly enriched for roles in covalent chromatin modification (*Setdb1*, *Auts2, Ctcf, Hdac2/5/8/9, Hist1h1d*), histone and DNA methylation (*Jmjd6*, *Kdm4a*) and deubiquitination (*Usp22*, *Kat2a*) (Fig. 2b, Supplementary Table TS8b). Intriguingly, the LC was a notable exception to the above trend in that it showed downregulation of genes from the same functional categories (*see Discussion*). IPA also highlighted fluoxetine-induced chromatin changes via the sirtuin signalling pathway (Fig. 2c), which is known to be energy-metabolism-dependent and represses transcriptional activity via deacetylation of histones, transcription factors and cofactors. Sirtuin signalling was differentially altered in most regions (18/27) and significantly repressed in a majority of these (13/18). Taken together, our results suggest that fluoxetine globally influences chromatin organisation by upregulating multiple genes involved in histone methylation, acetylation, and deubiquitination.

Lastly, we observed two globally downregulated DEG modules (module 1 and 2, Fig. 2b), which were enriched for membrane components (FDR *Q-val: 1.36e-4*), including ion channels (*Kcnj10, Kcna2, Cacna1i)*, membrane-bound receptors (*Calcrl, Erbb3, Nr3c2, Lrrtm2)*, solute transporters (S*lc6a11, Slc6a7)* and neuromodulators *(Adcy1, Slitrk2, Gabrg2, Epha6, Notch2)*. Inhibition of a number of these genes has been shown to exert therapeutic effects on depression, anxiety and other CNS disorders [81]. IPA analysis of region-wise DEGs highlighted brain-region-specific modulation of opioid, hippo, ephrin and dopamine signalling (Fig. 2c), suggesting potential molecular mechanisms for these known antidepressant and anxiolytic effects of SSRIs [82].

In summary, our bulk RNA-seq results indicate that chronic fluoxetine administration triggers profound and complex gene expression changes across the entire brain. These include major alterations in oligodendrocyte- and neuron-specific genes, as well as genes involved in energy production, chromatin modification and diverse pathways beyond serotonin.

### Genome-wide H3K27ac landscape of Fluoxetine action

MDD is marked by reduced histone acetylation in hippocampus and PFC [31]. Consistently, histone deacetylase inhibitors, which increase global histone acetylation levels, show strong antidepressant-like activity [83]. To complement our multi-regional transcriptome map, we used ChIP-seq to profile genome-wide fluoxetine-induced histone acetylation changes in each of the 27 brain regions. In this analysis, we targeted H3K27ac, the most well-studied acetylation signature of active regulatory elements such as enhancers and promoters [84]. We used DFilter (Supplementary Methods) to call a consensus set of 48,006 H3K27ac ChIP-seq peaks across the 27 regions—these peaks represent potential gene regulatory elements. Reassuringly, as in the case of RNA-seq, the average chromatin profiles of the 27 brain regions clustered primarily based on anatomical proximity (Supplementary Fig. S7a). However, the profiles of treated and untreated samples were clearly distinct (Supplementary Fig. S7b). Remarkably, 4511 peaks within the consensus set showed significant differential acetylation between sham and fluoxetine-treated animals in at least one brain region (abs log_2_FC ≥ log_2_ (1.25), FDR *Q-val* ≤ 0.1, Supplementary Table TS10, Supplementary Methods), indicating strong chromatin modulation by fluoxetine.

**Fig. 3:**
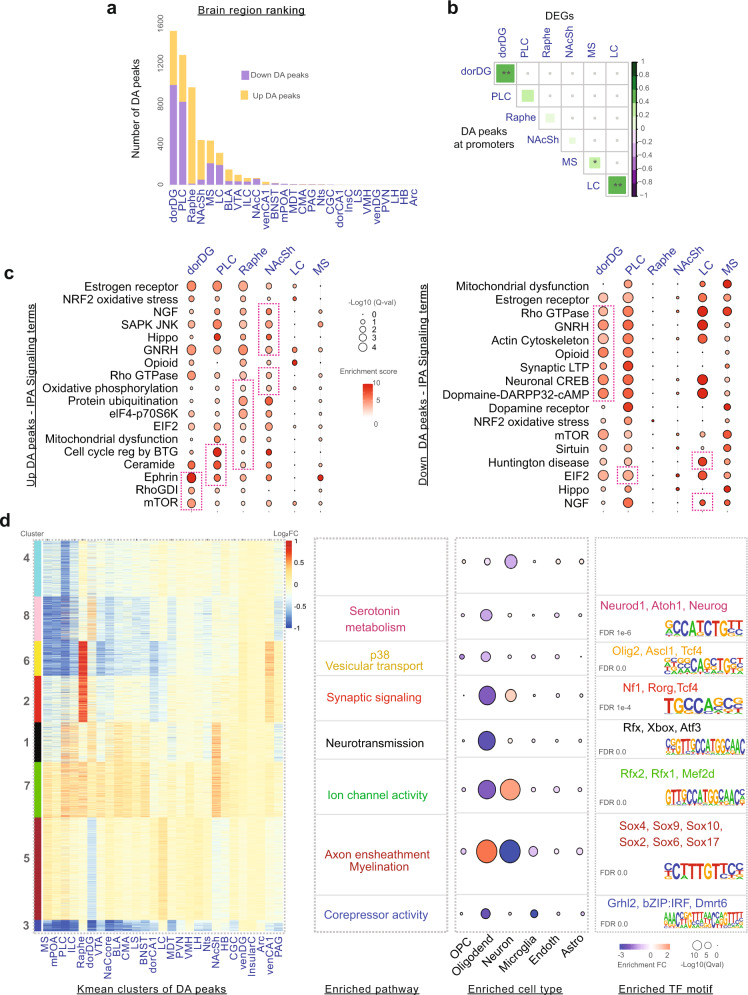
Histone acetylome-wide status of fluoxetine action. **a** Statistics of ChIP-seq dataset: Number of significant differentially acetylated (DA) peaks in the consensus set (FDR *Q-val* ≤ 0.1). Brain regions are ranked by the number of DA peaks. **b** Spearman correlations of DEG log_2_ fold-changes and their associated promoter DA peak log_2_ fold-changes in the top 6 brain regions (ranked by number of DA peaks). The intensity of the green denotes a higher correlation value as coloured in the scale bar, **: correlation *P-val* ≤ 0.01, *: *P-val* ≤ 0.05. **c** GREAT enrichment of region-wise DA peaks for IPA pathways in the top 6 brain regions as in **b**. Magenta boxes indicate pathway terms that were also enriched in the corresponding region-wise DEG set. Dot size indicates FDR *Q-val* and the colour denotes enrichment. **d** Functional annotation of the eight DA co-regulated gene clusters: top enriched pathways, enriched cell types and HOMER transcription factor motifs of each cluster are listed (FDR *Q-val* ≤ 0.1).

Amongst the 27 regions, the dorDG, PLC, raphe, NAcSh, MS and LC showed the most striking differential acetylation between fluoxetine-treated and control groups (Fig. 3a, Supplementary Table TS11). Importantly, raphe, NAcSh and LC were among the six top-ranked brain regions for differentially acetylated peaks as well as DEGs (Fig. 1d), suggesting that these were the most prominent fluoxetine-responsive regions. Of the six brain regions listed above, the dorDG and LC showed the greatest correlation in fold-change between DEGs and their corresponding differentially acetylated promoter peaks (*R* = 0.59 and 0.55 respectively; correlation *P-val* < 0.01 for both, Fig. 3b). As noted in multiple previous studies, the correlation between differential acetylation and expression is diminished by multiple factors, including post-transcriptional regulation, DNA methylation, presence of poised promoters, and measurement noise [38, 85]. Indeed, out of top 14 regions ranked by differential peaks, seven had higher correlations while the other seven brain regions showed lower correlation (*P-val* > 0.05; Fig. 3b, Supplementary Table TS12).

To examine the functional correlation between differentially acetylated peaks and DEGs, we applied the GREAT algorithm to IPA gene sets associated with fluoxetine-responsive DEGs (Fig. 3c, Supplementary Methods). Differentially acetylated peaks in the top six regions were enriched for cellular functions such as protein metabolism (eIF2 signalling, eIF4 signalling, ubiquitination), energy production (mitochondrial dysfunction, oxidative phosphorylation, mTOR signalling) and modulation of neuronal signalling (Rho-, opioid-, ceramide- and dopamine-signalling pathways). A substantial number of IPA annotations associated with DEGs were also associated with genes near differentially acetylated peaks (magenta boxes, Fig. 3c, Supplementary Methods). For example, in dorDG, multiple pathways enriched in downregulated differentially acetylated peaks were also identified as repressed in RNA-seq, and vice versa (magenta boxes, Fig. 3c). These trends were also evident among upregulated peaks in raphe and NAcSh. These results suggest strong functional consistency between differentially acetylated peaks and DEGs.

To identify and characterise differentially acetylated peaks modulated by fluoxetine in a similar manner across brain regions, we used k-means clustering to cluster differentially acetylated peaks by their fold-change profiles (Fig. 3d). For each cluster of differentially acetylated peaks, we identified enriched functional categories (GREAT, Supplementary Methods), transcription factor (TF)-binding motifs (HOMER, Supplementary Methods) and cell-type-specific markers (BRETIGEA database, Supplementary Methods). As in the case of DEGs, we observed two differentially acetylated clusters (Clusters 1 and 7) upregulated in >8 brain regions that were significantly enriched for ion channels (FDR *Q-val*: *5.8e-4*), synaptic signalling (FDR *Q-val: 0.01)* and neuron-specific markers (FDR *Q-val: 1.1e-14)* (Fig. 3d, Supplementary Tables TS13,14). Importantly, TF motifs of Rfx1/2, Mef2d and Atf3, all of which are implicated in depression and anxiety [86], were enriched in this cluster (Fig. 3d, Supplementary Tables TS15). Cluster 3, representing co-regulated differentially acetylated peaks globally downregulated by fluoxetine, was enriched for RNA polymerase II transcription corepressor activity (FDR *Q-val: 5.8e-4)* and for TFs associated with co-repressor functions (Grhl2, Dmrt6, Bzip:Irf). Cluster 5 consisted of differentially acetylated peaks upregulated in LC and downregulated in dorDG. This cluster showed strong enrichment for functional categories and cell type markers specific for oligodendrocytes. Interestingly, HOMER identified the Sox family of TFs, which are known to be dysregulated in MDD [87], as the most strongly enriched in Cluster 5.

Overall, the above results indicate consistency between differentially regulated pathways inferred from RNA-seq and ChIP-seq, namely energy and protein metabolism, ion channels and synaptic transmission. Analysis of histone acetylation changes in response to fluoxetine suggests the presence of distinct gene regulatory modules altered in neurons and glia, and provide candidate TFs that may contribute to these changes.

### Single-cell transcriptome analysis of the hippocampal DG

**Fig. 4:**
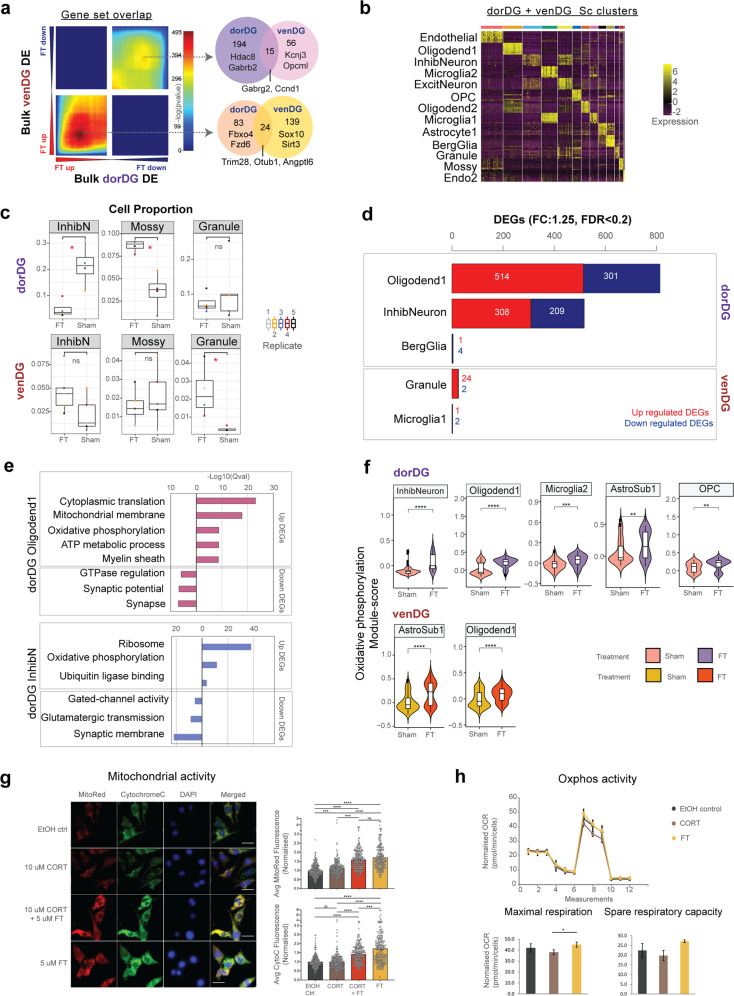
Cellular landscape of fluoxetine action in dorDG and venDG. **a** Rank-rank hypergeometric overlap (RRHO) maps show the threshold-free differential expression comparison between dorsal and ventral DG in this study. Pixels represent the overlap between the transcriptome of each comparison, with the significance of overlap (–log_10_ (*P-val*) of a hypergeometric test) colour coded. Genes along each axis are sorted from most significantly up-regulated (lower left) to most down-regulated (top right). Venn diagram shows overlap of significant DEGs in dorDG and venDG. **b** Heatmap showing 12 cell types obtained by clustering 3,517 single cells from 5 replicates per treatment group. Two-step clustering using supervised RCA2 clustering, followed by Seurat unsupervised clustering was performed. Top 10 markers specific for each cluster are plotted. **c** Cell types with significant changes in proportion between Sham and FT groups in dorDG or venDG (both regions shown to highlight region-specific differences). *: Wilcoxon *P-val* ≤ 0.05. **d** Treatment-specific DEGs (FT vs Sham) for dorDG and venDG cell types. Single cells from each cell type were aggregated by replicate into an averaged pseudo-bulk expression profile. Pseudo-bulk expression profiles were then used to calculate DEGs (absolute log_2_FC ≥ log_2_(1.25), FDR *Q-val* < 0.2). **e** Top GO terms following gene set enrichment analysis of cell-type-specific pseudo-bulk DEGs (FT vs Sham). **f** Module-score analysis for the oxidative phosphorylation gene set in dorDG and venDG cell types. Cell types with significant FT vs Sham module score are plotted. **** indicates: FDR *Q-val* < 3e-05, ***: FDR *Q-val* < 5e-03, **: FDR *Q-val* < 0.05. **g** *Left*, representative images of CRL-2199 glial line treated with vehicle control, corticosterone (CORT), fluoxetine (FT) and CORT + FT, stained with DAPI (blue) and MitoTracker Red (red) and immunostained against cytochrome c (green). Scale bars, 20 μm. *Right*, quantification of average MitoTracker Red (top) and cytochrome c (bottom) fluorescence signals normalised to vehicle control (*n* = ~200 cells per group using three independent experiments; values for individual cells are shown as open circles; error bars denote SEM). SEM, standard error of the mean. **h** Oxygen consumption rate (OCR) measurements normalised to cell number measured by Hoechst 33342 fluorescence signal (mean of 3 biological replicates ±SD, technical replicates =34). SD, standard deviation.

Thus far, we have used bulk-sample omics assays to characterise fluoxetine response in 27 brain regions. Though bulk-based assays are efficient for large-scale sample processing, they cannot resolve the heterogeneity of cellular responses within a tissue. We therefore complemented the above-described assays with scRNA-seq analysis of two brain regions. In this analysis, we prioritised the dorDG, which ranked first among the 27 regions in terms of the number of differentially acetylated peaks and third in the composite ranking based on the sum of log-ranks in ChIP-seq and RNA-seq (Supplementary Tables TS12). For comparison, we also included the venDG, a region at the other end of the hippocampus that shared 39 DEGs with dorDG. Note however that the majority of DEGs were distinct in these two regions (Fig. 4a). The relevance of these two regions was further supported by the fact that they have been widely implicated in MDD and antidepressant response [88–91].

We generated single-cell gene-expression profiles from dorDG and venDG micro-punches (*n* = 5 replicates, each replicate pooled from three animals). After stringent QC (Supplementary Methods, Supplementary Fig. S8), we retained 3,517 high quality cells for further analysis. Using supervised and unsupervised clustering of single cell transcriptomes [92, 93], we identified 12 distinct cell types in dorDG and venDG (Fig. 4b, Supplementary Fig. S9, Supplementary Tables TS16–17*and* Supplementary Methods). We hypothesised that fluoxetine may alter cell type proportions as well as cell-type-specific gene expression, and that these alterations may differ between the two dentate gyri. Indeed, we observed a substantial increase in granule cell counts in the fluoxetine-treated venDG (*P-val*: *0.03*, Fig. 4c, Supplementary Tables TS18). This cellular phenotype of treated animals could potentially have behavioural consequences, since increased granule cell proliferation has been observed in the ventral hippocampus of antidepressant responders and stress-resilient animals [94, 95]. Notably, no such shift was observed in dorDG (Fig. 4c). Rather, in dorDG, fluoxetine drove a significant decrease in the number of inhibitory neurons relative to control and an increase in mossy cells (*P-val*: 0.03 in both cases). The fluoxetine-induced shifts observed in dorDG could also have behavioural consequences, since mossy cell depletion is associated with increased anxiety in rodents and inhibitory neuron abundance is positively associated with MDD [96, 97]. In summary, these results highlight distinct cell types in these two hippocampal brain regions that could be prioritised in studies of the mechanism of action and therapeutic effects of SSRIs.

Next, we sought to identify the cell types whose transcriptomes were substantially altered by fluoxetine, i.e. the cell types with a substantial number of DEGs (Supplementary Tables TS19, Supplementary Methods). In the dorDG, two cell types contributed the vast majority of DEGs: oligodendrocyte1 (514 up, 301 down) and inhibitory neurons (308 up, 209 down) (Fig. 4d, Supplementary Tables TS19). Notably, a substantial number of DEGs were shared between these two cell types (148 up, 47 down). Although DEGs may be detectable in additional cell types in larger cohort sizes, this result nevertheless suggests that these two cell types may be the strongest responders in dorDG. The transcriptional response of the venDG was more modest (Fig. 4d, Supplementary Tables TS19). The finding that fluoxetine substantially alters gene expression in oligodendrocytes in dorDG constitutes yet another link to previous results on depression, namely that glial cell types play a prominent role in the etio-pathology of MDD [45, 78].

To link cell-type-specific DEGs to known biological pathways, we tested for enriched gene annotations (FDR *Q-val* ≤ 0.05, Fig. 4e, Supplementary Tables TS20 and Supplementary Methods). In both oligodendrocyte1 cells and inhibitory neurons, upregulated DEGs showed strong enrichment for cytoplasmic protein translation machinery (*Rps3, Rpl18, Rpl23a*), ETC complex and ATP metabolism genes (*Uqcrh, Uqcrq, Ndufsv2, Atp5f1d, Cox6*) (Fig. 4e). DEGs downregulated in oligodendrocyte1 were enriched for synapse and GTPase regulators (*Gabrb1, Gria2, Gria3, Syt1*), while those in inhibitory neurons were enriched for ion channels and glutamatergic transmission (*Cacna1a, Nlgn2, Epha7, Ntrk2, Gria2*). Importantly, these terms were strongly concordant with the broad trends inferred from bulk RNA-seq and ChIP-seq (Figs. 2c, 3c). Next, we used a gene-module-scoring approach (Supplementary Methods) to examine in a hypothesis-driven manner if these gene sets might show systematic upregulation in additional cell types. Notably, the oxidative phosphorylation module score was significantly increased by fluoxetine administration in 5 of 12 dorDG cell types (oligodendrocyte1, inhibitory neurons, astrocyte1, oligodendrocyte precursor cells and microglia2) and in oligodendrocyte1 and astrocyte1 cells in venDG (FDR *Q-val* ≤ 0.01, Fig. 4f, Supplementary Tables TS21). These results consistently suggest that increased energy metabolism may constitute a central mechanism of action of fluoxetine, and that glial subtypes (oligodendrocyte1, astrocyte1 and microglia2) could be potential mediators of this effect.

We examined if these transcriptomic changes would result in functional alterations in mitochondria and oxphos activity. To assess the mitochondrial membrane potential gradient generated as a result of oxidative phosphorylation, we treated the glial line CRL-2199 with FT, corticosterone (CORT, a glucocorticoid stressor) and corticosterone+fluoxetine (CORT + FT) for 3 days (Supplementary Methods). Following the different treatments, we labelled live cells with MitoTracker Red, a dye that localises to the mitochondrial membrane in a membrane potential-dependent manner, and immunostained for the oxphos membrane protein cytochrome c. Quantification of the average fluorescence signal revealed that MitoTracker Red was significantly upregulated in the fluoxetine group by 1.7-fold relative to vehicle control (*P-val* < 0.0001) and by 1.6-fold in the CORT + FT group (*P-val* < 0.0001, Fig. 4g, Supplementary Methods). This suggests an increase in mitochondrial abundance. Similarly, there was a 1.7 and 1.4-fold increase in cytochrome c levels in the FT and CORT + FT group compared to vehicle, respectively, suggesting a fluoxetine-induced increase in oxphos activity. Next, we measured the oxygen consumption rate (OCR) of cells as a proxy for the oxidative phosphorylation rate. In the presence of a stressor, the OCR decreased, whereas fluoxetine administration increased oxygen consumption by 11 percent relative to the CORT group (*P-val*: 0.04, FT vs CORT, Fig. 4h), indicating a shift towards higher oxidative phosphorylation in glial cells. Taken together, these assays show that fluoxetine upregulates mitochondrial abundance and mitochondrial ATP pathways in vitro, which corroborates with the concerted transcriptomic and epigenomic regulation of energy metabolism genes as revealed by the bulk and single-cell analysis.

**Fig. 5:**
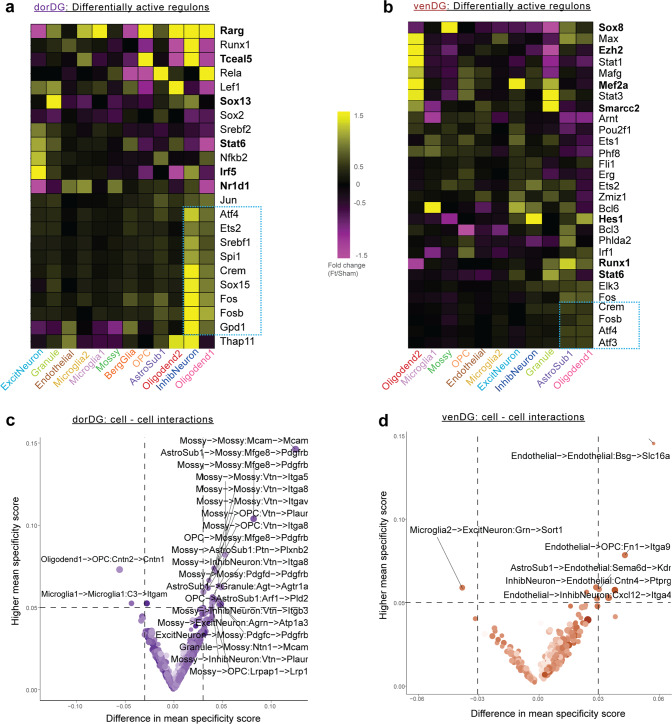
Association of dorDG and venDG cell types with master regulators and cellular cross-talks. **a** Heatmap of differentially active regulons identified by SCENIC in dorDG cell types, coloured by fold-change of regulon activity score in FT vs sham. Blue rectangle indicates regulons related to energy metabolism. Bold indicates the top differential regulon for major cell types. **b** As in **a**, but for venDG. **c**. NATMI-identified differential ligand-receptor pairs in dorDG cell types between FT and Sham. Top differential interactions (by higher specificity score and greater difference in specificity score) are labelled. **d** As in **b**, but for venDG.

We next sought to explain the above-described patterns of differential expression by identifying cell-type-specific ‘regulons’, which are defined as TFs coupled with their downstream targets. Using SCENIC (Supplementary Methods), we detected 178 regulons in dorDG and 173 in venDG (Supplementary Tables TS22a, Supplementary Fig. S10), of which 136 and 131, respectively, were differentially active in at least one cell type (FDR *Q-val* < 0.1, Fig. 5a, Supplementary Tables TS22b). Consistent with the sharing of DEGs between oligodendrocyte1 and inhibitory neurons in dorDG, we identified a shared set of fluoxetine-activated regulons (*Atf4, Jun, Crem, Fosb, Ets2, Sox15, Srebf1, Fos, Spi1*) in these two cell types (blue box, Fig. 5a). These transcription factors drive ATP synthesis, as well as transcription and translation of cytoprotective genes, in response to mitochondrial signalling [98, 99]. Intriguingly, the regulons of four of the above-mentioned TFs (*Crem, Atf4, Fos, Fosb*) were also activated by fluoxetine in venDG astrocyte1 and oligodendrocyte1 cells, albeit less strongly (Fig. 5b). These are precisely the venDG cell types in which the oxidative phosphorylation gene module was upregulated (Fig. 4f). Taken together, these results suggest that the above-mentioned TFs may contribute to hippocampal activation of energy metabolism genes in response to fluoxetine.

In addition to treatment-specific regulatory programs, we investigated fluoxetine-induced changes in signalling between pairs of cell types. We performed signalling analysis using NATMI (Supplementary Methods), which leverages a literature-curated catalogue of ligand-receptor pairs to estimate their interaction strength in pairs of cell types. By comparing fluoxetine-treated vs. control samples, we detected 22 ligand–receptor combinations with differential interaction scores in dorDG and 6 in venDG (Fig. 5c, d, Supplementary Tables TS23, Supplementary Fig. S11). Interestingly, mossy cells were prominent in dorDG, participating in 18/20 signalling interactions with higher scores in the fluoxetine-treated group (Fig. 5c, d). Of these, *Pdgfrb* stood out as the receptor with the largest number of differential interactions (5/20 interactions). SSRI treatment in vitro activates *Pdgfrb* via serotonin receptors *(Htr1a, Htr2b)*, resulting in increased neuroprotective growth factor signalling [100]. In addition, mossy cells have an established role in adult neurogenesis [101, 102] and mediate anti-anxiolytic and neurogenic responses to antidepressants [103]. Together with the observed increase in mossy cell abundance (Fig. 4c), these results suggest that mossy cells could be an important mediator, and provide candidate signalling interactions that may contribute to the mechanism of action of fluoxetine.

In summary, scRNA-seq analysis of the two hippocampal sub-regions highlights 1) the substantial differences between dorDG and venDG in their molecular responses; 2) increased energy metabolism as a prominent mechanism of fluoxetine action; and 3) glial subtypes, inhibitory neurons and mossy cells as cellular effectors of fluoxetine.

## Discussion

Here we mapped the transcriptomic and epigenomic landscape of chronic fluoxetine exposure across the rodent brain. Prior studies examined fluoxetine-mediated genome-wide transcriptional alterations in limited brain regions using microarrays [22, 23, 104, 105] or targeted profiling of candidate genes [106]. Our work expands current understanding of fluoxetine action by investigating a broader panel of 27 brain regions, adopting a multimodal approach of RNA-seq, H3K27ac ChIP-seq profiling, and complementary scRNA-seq of two hippocampal regions. The unique breadth of our study enabled comprehensive insights into fluoxetine action including: a) the occurrence of thousands of region-dependent molecular changes across the brain, a majority of which are previously unknown; b) identification of the raphe, nucleus accumbens (NAc), dorsal dentate gyrus (dorDG), locus coeruleus (LC) and pre-limbic cortex (PLC) as the most strongly affected regions; c) increases in chromatin remodelling, energy metabolism and mitochondrial gene expression; d) cell-type-specific changes in oligodendrocyte and neuronal subtypes; and e) stark differences in fluoxetine response along the dorso-ventral axis of the dentate gyrus.

**Fig. 6:**
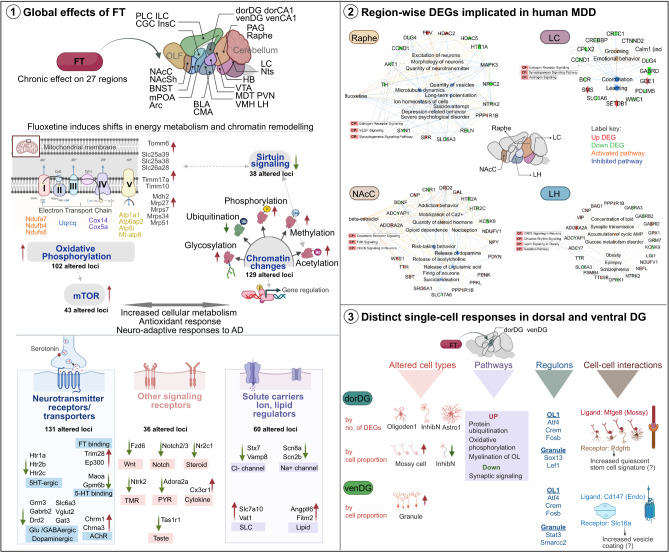
Schematic summary of effects of chronic fluoxetine identified in this study. **1**. Broad effects of chronic fluoxetine: Enriched functional terms and overarching pathways. **2**. DEGs across brain regions showed significant enrichment for MDD associated gene loci. A network diagram for 4 regions and their respective MDD-DEGs are illustrated. Region specific downstream pathways and functional implications are shown. Red – upregulated gene, green- downregulated gene; Orange terms indicate upregulated functional activity, while blue denotes inhibition of the functional term. **3**. Fluoxetine-induced effects at single-cell resolution.

Fluoxetine treatment produced profound changes in transcription and chromatin openness across multiple regions of the brain. We identified 4447 transcripts and 4511 peaks that underwent alterations in at least one brain region following fluoxetine treatment (Figs. 1d, 3a). Of these, we observed significant enrichment of DEGs for single nucleotide polymorphisms identified in GWAS studies for MDD, SSRIs and antidepressant response (Fig. 1g, Supplementary Tables TS5). This study therefore expands the list of MDD-informative brain regions that warrant modelling in animal studies of stress and antidepressant mechanisms. Notably, several region-wise DEGs that coincided with GWAS and PsyGeNET loci (e.g. *Opkr1*, *Kcnk9*, *Sst*, *Slc6a3*, *Slc5a7*, *Slc7a10*, *Negr1*) have been investigated as druggable targets for improving the efficacy and safety of neuropsychiatric drugs [107, 108] (Fig. 6). Moreover, 58 differentially regulated transcripts identified in this study overlapped candidates from three gene expression studies of MDD [45, 109] (Supplementary Tables TS24), a vast majority of which were altered in multiple regions beyond the single region profiled in the respective human studies (e.g. *Arhgef25*, *Kmt2a*, *Mettl9*, *Rhoa*, *Mgat4c)*. Consistent with this, we observed a good overlap of transcriptional changes between our datasets and antidepressant responses in multiple stress paradigms. We also identified specific cell types in which known MDD genes were altered by fluoxetine (e.g. *Dock4* in dorDG oligodendrocyte1, *Prkar1b* in venDG granule and *Klf26b* in inhibitory neurons) (Supplementary Tables TS24). These analyses highlight the relevance of fluoxetine-induced alterations identified in this study to human clinical phenotypes of MDD and treatment response, and reveal additional brain regions, gene candidates and cell types for further investigation.

Our composite ranking of the 27 brain regions, based on the sum of log-ranks in ChIP-seq and RNA-seq (Figs. 1d, 3a, Supplementary Tables TS4), revealed raphe, NAcSh, dorDG, LC, NAcC and PLC as the regions with the strongest molecular response to fluoxetine. The NAcSh and LC showed the next strongest accumulation of transcriptomic and epigenomic changes, contrary to a previous microarray study that detected merely 39 DEGs in LC and ranked the region’s fluoxetine response as low [22]. Though biochemical studies [110–112] have highlighted that neurotransmitter levels in the LC and NAc regulate fluoxetine-induced behavioural responses, a map of the underlying transcriptomic and epigenetic correlates has been missing hitherto. The extensive alterations in multiple receptor-driven signalling pathways (Fig. 6) across multiple regions, could explain molecular adaptations leading to the therapeutic and side effects of chronic fluoxetine regimes.

To examine the biology underlying these antidepressant-induced gene regulatory changes, we identified pathways and co-regulated network modules enriched in differentially expressed genes and acetylated peaks (Figs. 2a–c, 3c, d). We found evidence for functional consistency between DEGs and differentially acetylated loci. Functional enrichment analysis of k-means cluster modules and region-wise pathway enrichment identified chromatin remodelling, cellular metabolism and mitochondrial themes across most regions.

Fluoxetine drove an overall increase in the transcription of genes involved in energy production. MDD patients show both reduced brain glucose metabolism and mitochondrial impairments [113–116]. Interestingly, antidepressant treatments normalised some of these dysregulated proteins and reversed depressive behaviour [117–120]. The >100 DEG and DA loci we identified in this functional category form an unprecedented candidate list of potential SSRI-induced energy metabolism regulators (Fig. 6). Of the energy metabolism DEGs, upregulation of *Sdhb*, *Mdh2*, *Cox5a, Pfkl, Ck and Aacs* transcripts in specific hippocampal subregions is in agreement with their increased activity or protein levels in response to antidepressants [118, 121, 122]. We observed such changes in diverse additional regions (>9) beyond the hippocampus.

In addition to mitochondrial alterations, we found widespread regulation of histone modifications and chromatin signatures (Fig. 6). Studies have shown that chronic stress and depression reduces H3 histone methylation, resulting in deregulation of neuronal plasticity [123]. It has been suggested that antidepressants reverse these chromatin alterations, although these reports are largely limited to modifications at specific gene promoter loci and single brain regions [123–125]. Here, we find that fluoxetine pervasively influences chromatin permissiveness by regulating the expression of a gamut of genes involved in histone methylation, phosphorylation and deubiquitination. Together with AD-induced global increases in energy metabolism, these changes in chromatin remodelling could synergistically drive transcriptional cascades involved in neurotransmitter and ion transport, vesicular trafficking, protein synthesis, protein folding and clearance [126]. Antidepressant induced chromatin changes have also been shown to resemble epigenetic signatures seen in stress-resilient animals [127]. We propose that further investigation of our genome-wide candidate loci could potentially reveal fundamentally novel AD and stress resilience mechanisms.

We then examined specific cell types associated with fluoxetine response. We found that oligodendrocytes and neurons were the two major fluoxetine-responsive cell types in our analyses, however there was a strong heterogeneity across the 27 brain regions (Supplementary Fig. S5b). Interestingly, oligodendrocyte subtypes and a subset of the DEGs we identified have been implicated in a recent single-cell study on the PFC in MDD [45] (Supplementary Tables TS24). Our scRNA-seq data from dorDG and venDG provided a higher resolution map of fluoxetine-induced effects and their regional differences: five cell types in dorDG and 2 in venDG showed a significant increase in oxidative phosphorylation scores and shared relevant upstream regulators (Figs. 4f, 5a, b). Taken together, these five hippocampal cell types could be prioritised for further investigations of SSRI-induced metabolic changes. We propose that ligand-receptor interactions involving mossy cells (*Pdgfrb*, *Megf8/Vtn*) could be important signalling mediators of fluoxetine action in dorDG (Fig. 5c), and promising candidates for follow-up studies.

Studies on differences in antidepressant efficacy between males and females have led to inconclusive findings [128]. While some studies have reported sex-dependence of antidepressant-induced behavioural and molecular changes [129, 130] others have concluded that some changes are sex-independent [131, 132]. Due to the known influence of variations in the female rat’s oestrus cycle on fluoxetine’s efficacy [133, 134] and the additional resources and handling associated with syncing the oestrus phase of a large cohort, we chose to focus our study on male rats. Future studies are needed to investigate sexual dimorphism of fluoxetine’s response across diverse brain regions to complement the current dataset [135] leveraging the region-specific effects reported here.

In summary, our results greatly expand the current understanding of the spatial molecular complexity of fluoxetine response. This dataset highlights understudied brain regions and provides a framework for selecting candidate genes, pathways and cell types for further mechanistic analysis and identification of targetable pathways for depression and anxiety.

## Data and codes availability

Raw data have been deposited at the NCBI’s Gene Expression Omnibus, under the following accession numbers: ChIP-seq- ***GSE193040***; RNA-seq- ***GSE194289;*** scRNA-seq- ***GSE197622***. Codes for most of the routines are available at Github (https://github.com/arulrayan/Integrative-multi-omics-landscape-of-fluoxetine-action-across-27-brain-regions) or upon reasonable request.

## Supplementary information

Supplementary Methods

Supplementary Figures

Supplementary Tables
