# Supplementary material for: Integrative multi-omics landscape of fluoxetine action across 27 brain regions reveals global increase in energy metabolism and region-specific chromatin remodelling
Source: Mol Psychiatry. 2022 Sep 2;27(11):4510–25. doi: 10.1038/s41380-022-01725-1 (PMC9734063; doi:10.1038/s41380-022-01725-1)
Supplement: Supplementary file 1 — Supplementary Methods [file 41380_2022_1725_MOESM1_ESM.pdf]

Supplementary Methods:

Animal housing and treatment

All procedures were performed in accordance with the guidelines established by the Canadian Council on Animal Care (CCAC) with protocols approved by the McGill University Facility Animal Care Committee (FACC). Long-Evans rats were purchased from Charles River (RRID:RGD_2308852) and bred at the Douglas Mental Health University Institute animal facility. Offspring were weaned on postnatal day 22. 60-day old uncharacterized male rats were housed in pairs and separated as control (Sham) group and fluoxetine-treated (FT) group. Food and water were provided *ad libitum* and bedding was changed weekly. Body weights were collected at the start of treatment and on the sac date. Fluoxetine (18 mg/kg/day) was provided *ad libitum* in the drinking water. This formed the treatment group rats, while rats in the Sham group received only water. Fluoxetine dosing was controlled by estimating the water consumption. We first estimated the baseline consumption by measuring the 24h water intake of the animals. To ensure a dose of 18mg/kg/day (bw), the fluoxetine concentration in the water was individually adjusted per cage according to the baseline consumption and the weight gain per week. During the six-week treatment, the animals were weighed weekly, and water consumption was measured every 48 hours, three times a week. The bottles containing the solutions were covered in aluminium foil and placed on a plexiglass sheet within the cage top. Rats were pair housed. Fluid consumption was measured every Monday, Wednesday and Friday and the solutions were replaced in new water bottles at this interval. Animals of both Sham and FT group were sacrificed after 6 weeks, between 10:00 am and 12:00 pm.

Behavioural scoring – Forced Swim Test

During day 1 (training or acclimatization session) rats were placed individually into a clear plexiglass cylinder (25cm diameter, 65cm height) with water at a depth of 35-40 cm (23-25°C) for a period of 15 min. This session was recorded using a video camera and the total time the rat spent immobile or swimming was measured.

Day 2 (test day, 24-hr post-training session): The training phase and the testing phase of the experiment were completed at the same time of day for each rat. The test session lasted 10 min. Both training and test sessions were recorded using a digital camera kept at water level to observe the entire body length of the animal and assist in better coding the activity. Time spent immobile was defined as the period of time the animal spent motionless or floating, or only taking the minimal actions required to keep its head above water level. Swimming was measured as any coordinated motion of the animal in the container away from the walls. To ensure accuracy, immobility was calculated as follows: $T_{immobile} = 600s - (Time_{Climbing} +$

$Time_{Swimming}$). The time spent immobile in the FST was used as a measure of "behavioural despair" (***Figure 1b***, ***Supplementary Table TS1b)***.

Note: Forty rats per condition were used in the omics-data generation (bulk RNA-seq, ChIP-seq, and scRNA-seq assays), whereas these behavioral assays were performed on an independent cohort of 10 animals per group (Sham vs. FT). This strategy was employed to avoid any confounding effect on the transcriptome induced by the battery of behavioral tests conducted.

Tissue collection

Rat brains were rapidly removed, flash frozen using cold isopentane and stored at -80°C. We did a 200 µm thickness coronal slicing of the frozen brain. We then micro-punched different brain regions from these 200 µm sections, using 1 mm and 0.5 mm diameter tissue punchers, while maintaining -20°C cryostat chamber conditions. We chose the bregma planes of each of the 27 regions following the well-defined illustration found in Paxinos atlas [1], ***Supplementary Table TS1***. Tissue material for any particular region was pooled across 2-3 consecutive sections of the brain. Tissue punches of a region were pooled across 10 animals/brains to form one replicate. There were 4 biological replicates (40 animals per treatment group) for RNA-seq and 2 biological replicates (20 animals per treatment group) for ChIP-seq. In order to avoid introduction of batch effects/technical processing artefacts, corresponding Sham and FT samples of one replicate were processed in parallel.

Bulk RNA-seq data generation

Frozen, pooled brain tissue punches for each region were thawed on ice in 1 ml Trizol. Tissue was then homogenized using a manual glass douncer with 7-15 slow strokes on ice. RNA was extracted from each replicate sample using the Qiagen RNA Mini kit (Qiagen, Cat# 74104) as per the manufacturer's instructions with on-column DNase I treatment. RNA quality was examined using a Bioanalyzer 2100 (Agilent technologies, Santa Clara, USA). RNA Integrity Number (RIN) values ranged from 7.1 to 10, with a median value of 9.5. cDNA libraries were prepared using 300 ng of total RNA, from 27 regions in every replicate, using the Illumina TruSeq Stranded Total RNA LT set (Illumina, Cat# RS-122-2301). Paired-end, 76 bp read length RNA-seq was carried out on the Illumina HiSeq 2500 at a depth of 20M reads per sample.

RNA-seq data analysis

a) Mapping: RNA-seq data were aligned to the *Rattus norvegicus* rn5 genome (Rn5 ENSEMBL) with the STAR aligner. Read counts were concurrently calculated using HTseq. Any dataset that failed the default Fastqc sequencing parameters and < 75% mapping rate was discarded.

b) Visualisation using Principal component analysis (PCA) and t-Distributed Stochastic Neighbor Embedding (tSNE):

Each brain region had 8 datasets (4 Sham and 4 FT replicates). The RNA-seq read counts were quantile normalised, followed by log transformation. Batch correction was performed using limma, adopting default parameters with replicates specified as batches. Following normalisation and batch correction, the top 20% of genes (by read counts) were visualised using tSNE and PCA (***Supplementary Figure S1a***). (Note that the normalisation and batch correction described here were performed for visualisation purposes only.)

c) Outlier removal: Region-wise PCA plots (PC1 and PC2 loadings) of normalised read counts were reviewed for outlier detection. Outliers were defined as data points outside 1.5 standard deviations from the mean of both PC loadings (PC1 & PC2). Two biological replicates of CGC region were identified as outliers and removed from further downstream analysis (***Supplementary Figure S1b***).

d) Differential analysis (DEG calling):

DESeq2 (https://bioconductor.org/packages/release/bioc/html/DESeq2.html) was used for computing differential expression statistics between Sham and FT samples per region. We provided the original read count matrix as input for DEseq2, and used the recommended batch correction mode, wherein replicate numbers constituted batches. DEG cut-offs were as follows: absolute $\log_2 FC \geq \log_2 (1.25)$ and FDR $Q\text{-}val \leq 0.1$ (***Supplementary Figure S2, Supplementary Table TS4b***). All read counts of region-wise DEGs were also visualised by tSNE (***Supplementary Table TS2, Supplementary Figure S1)***.

Validation of DEGs

High-throughput qPCR

The expression levels of 16 top DEGs from six brain regions (8 upregulated and 8 downregulated genes per region; 96 in total) were assayed using high-throughput Fludigm BioMark™ HD System Real-Time PCR. Briefly, diluted cDNA samples from FT and Sham groups were mixed with reaction reagents (primers, RT enzymes) and loaded on microfluidic chips. Chips were read by the Fludigm BioMark™ HD System Real-Time PCR suite. qRT-PCR results were analysed using the formula, $2^{-\Delta Ct}$ (ΔCt = Target gene – Reference gene (*B2M*)), and fold changes were computed. We plotted qPCR fold changes of the 8 'Up' and 8 'Down' RNA-seq DEGs using box-plots, and calculated the statistical significance between the two

groups using Wilcoxon rank sum test (***Supplementary Figure S3a***). *P-val* < 0.05 was accepted as statistically significant.

RNAscope, Image Acquisition and Analysis

To validate the regional heterogeneity of DEGs, we used RNAscope on brain tissue sections covering 3-4 regions. *Otub1*, *Trim28* and *Sirt2* were the DEGs chosen for single molecule RNA validation. 10 μm-thick sections of frozen fluoxetine-treated and untreated rat brains were sectioned and stored at -80°C. RNAscope Multiplex Fluorescent V2 assay (ACDBio, Cat# 323100) was implemented following the manufacturer's instructions for fresh frozen samples. In summary, sections were fixed in 4% paraformaldehyde in 1x PBS at 4°C for 15 min and then dehydrated in increasing concentrations of 50%, 70%, 100% ethanol. The sections were treated with hydrogen peroxide for 10 min, followed by RNAscope Protease IV for 30 min, both at room temperature. The probes were diluted to a working concentration as specified by the manufacturer and then hybridized with the sections for 2 h at 40°C. Sections were stored in 5x SSC overnight before hybridization with AMP 1, AMP 2 and AMP 3 for 30 min, 30 min and 15 min respectively at 40°C. Fluorescent dyes used were Opal 520 (Akoya Biosciences, Cat# FP1487001KT), Opal 570 (Akoya Biosciences, Cat# FP1488001KT) and Opal 690 (Akoya Biosciences, Cat# FP1497001KT), diluted at 1:1000, 1:1000 and 1:750 respectively in TSA buffer. Sections were then incubated with HRP-C1 for 15 min at 40°C, with the Opal dye for 30 min at 40°C and lastly with HRP blocker for 15 min at 40°C. The incubation process for HRP-C1 was repeated for HRP-C2, HRP-C3 and HRP-C4. Slides were then briefly dried to remove excess liquid and stained with DAPI using NucBlue Fixed Cell ReadyProbes Reagent (DAPI) (Life Technologies, Cat# R37606). The sections were mounted using ProLong Gold Antifade Mountant (Life Technologies, Cat# P36930) and allowed to dry overnight.

Images were acquired using a Leica confocal microscope. The same laser settings were used for Sham and fluoxetine-treated samples. Quantification of RNA expression was done using Python. Briefly, the images were manually cropped to selected regions of interest. The images were then clipped at the 99.99th percentile and smoothed using a Gaussian blur. Quantification of fluorescence was calculated at a selected percentile threshold for the treated samples. The selected thresholds were usually between 95th to 99.5th percentile. The absolute value used for thresholding the fluoxetine-treated sample was then used for the paired untreated sample. The number of pixels above the threshold was treated as an indication of the level of expression. An estimate of the number of cells in the image was obtained by quantifying the DAPI fluorescence. The DAPI images were clipped at the 99.99th percentile, smoothed and thresholded using multi-otsu thresholding. An estimate of the mRNA expression per cell was calculated by taking the number of pixels above threshold for each mRNA divided by the number of pixels for DAPI (***Figure 1f, Supplementary Figure S3b-e***).

Functional enrichment analysis of DEGs

GWAS enrichment:

GWAS catalogue genes associated with five clinical phenotypes (SSRI response [2], broad antidepressant response [3], MDD [4], Alzheimer's [5] and alcoholism [6]) were used for this analysis. For every region, the proportion of GWAS catalogue genes within the list of DEGs was calculated. For each phenotype, we simulated a random gene set wherein the gene length was similar to that of the GWAS genes. For every region analysed, the ratio of the fraction of GWAS genes in the list of DEGs versus the fraction of GWAS genes in the list of the random gene set was calculated, and referred to as GWAS enrichment score. We re-ran the above step 100 times to make sure we obtained a fair distribution of random control genes. Two-proportion z-test was used to calculate the *P-val* of the difference in fractions (***Figure 1g, Supplementary Table TS5a***).

PsyGeNET enrichment:

To query the PsyGeNET database, which manually curates genes involved in multiple human cognitive disorders by literature search, we downloaded the PsyGeNET "all gene-disease associations file", available at http://psygenet.org/web/PsyGeNET/menu/downloads. For each disease, we first intersected the total number of disease-associated genes with our list of all rat genes. Enrichment of disease-associated DEGs within each region-specific list of DEGs was then calculated using Fisher's exact test. This process was performed on all brain regions; we corrected for multiple testing (Benjamini-Hochberg) per psychiatric disorder (***Supplementary Figure S5a***).

Rank-Rank hypergeometric analysis (***Supplementary Figure S6***):

To examine the overlap in gene expression changes between our dataset and prior results in the literature, we downloaded raw HTSeq counts from ([65], GSE81672) and raw microarray data from ([66], GSE43261). For Bagot et al., we focused on stress-susceptible imipramine responders versus stress-susceptible saline controls (to examine the effect of a successful imipramine response on prior-stressed animals) in three regions – amygdala, nucleus accumbens and prefrontal cortex. For Carazo-Arias et al., we considered the comparison between control and fluoxetine responders, as well as between control and all fluoxetine-treated animals (i.e. non-responders and responders).

For each data set, we used DESeq2 to re-analyze the raw counts for the designated comparison as the complete set of analyzed results was not made publicly available. To calculate enrichment in overlap, we used the rank-rank hypergeometric overlap (RRHO2) package (https://github.com/RRHO2/RRHO2), ranking all genes found in both the external dataset and our dataset by the degree of differential expression

($-\log_{10}$(*P-val*) x effect size direction), and correcting all values by Benjamini-Hochberg. For Bagot et al., we ran the RRHO2 analysis on all corresponding anatomically equivalent regions: for amygdala, we calculated the RRHO overlap for the basolateral amygdala (BLA) and central medial amygdala (CMA); for the nucleus accumbens, we calculated the RRHO overlap for the nucleus accumbens core (NAcC) and nucleus accumbens shell (NAcSh); for the prefrontal cortex, we calculated the RRHO overlap for the cingulate cortex (CGC), infralimbic cortex (ILC) and prelimbic cortex (PLC).

Cell type enrichment:

To test the enrichment of DEGs for cell type specific markers, we used the BRETIGEA database (https://github.com/andymckenzie/BRETIGEA) that provides a marker list for each of the following cell types – neurons, oligodendrocytes, microglia, oligodendrocyte progenitor cells (OPC), astrocytes and endothelial cells. For each cell type, we calculated the enrichment of cell type-specific DEGs (for up and downregulated DEGs) within the provided list of cell type-specific genes (against a background of all genes in the database) using Fisher's exact test. This process was performed on all brain regions; we corrected for multiple testing (Benjamini-Hochberg) per cell type ***(Supplementary Figure S5b, Supplementary Table TS7)***.

Co-regulated gene clustering and pathway enrichment:

We constructed the $\log_2$FC matrix over the union set of DEGs across 27 brain regions. This matrix was z-transformed and used as input for k-means clustering (no. of clusters=9). We used anRichment to perform gene set enrichment within a larger, comprehensive collection of GO, HDSigDB, MSigDB and several well-curated neurogenomics gene sets. anRichment is available as an R function:

(https://horvath.genetics.ucla.edu/html/CoexpressionNetwork/GeneAnnotation/). In addition, we also ran enrichment using GOrilla (http://cbl-gorilla.cs.technion.ac.il/). To test enrichment for each k-means module, DEGs in that module constituted the foreground set, while the union of DEGs in the remaining modules served as the background set. Gene categories with enrichment ratio ≥ 1.5 and FDR *Q-val* ≤ 0.01 were retained. Additionally, we discarded the enriched gene categories if there were less than 5 DEGs in that category. Redundancy between two functional categories was defined as overlap of ≥ 40% in the number of DEGs. To display the non-redundant significantly enriched gene categories in ***Figure 2a***, we further selected the top non-redundant category terms from anRichment and GOrilla results. Almost all enriched terms overlapped in both anRichment and GOrilla results. The complete anRichment results and cluster 3 GOrilla results are listed in ***Supplementary Table TS8a***.

Gene Set Enrichment Analysis (GSEA):

We ran GSEA using the 'Run GSEA Preranked' tool. For each region, genes were first sorted according to their log_2FC values (positive or negative), and then ranked according to their FDR values by taking the inverse of the FDR (i.e., we calculated 1/FDR for genes with positive log_2FC values, and -1/FDR for genes with negative log_2FC values). The ranked gene list was then passed into GSEA. GO Biological Process terms (c5.go.bp.v7.5.1.symbols.gmt, http://www.gsea-msigdb.org/gsea/downloads.jsp; ***Figure 2b***) or GO Molecular Functions terms (c5.go.mf.v7.5.1.symbols.gmt, http://www.gsea-msigdb.org/gsea/downloads.jsp; ***Supplementary Figure S4***) were loaded as the reference GSEA database. The following settings were used: number of permutations = 500, enrichment statistic = classic, max gene set size = 400, min gene set size = 5, normalization mode = meandiv, omit features with no symbol match = true, seed for permutation = 123. Following GSEA, we set a FDR threshold of 0.1 to denote significance (***Figure 2b, Supplementary Figure S4, Supplementary Table TS8b***).

Ingenuity Pathway Analysis (IPA):

IPA (www.ingenuity.com) was used to explore and compare the relationship among the region-wise DEGs. The log_2FC and FDR matrix of region-wise DEGs, along with the gene identifiers (RefSeq gene names) were uploaded into IPA. The canonical pathway tool was used to identify the top pathways associated with DEGs between compared conditions. The "Expr log Ratio" option and default IPA settings (absolute log2FC > 0.32 and FDR < 25%) were used to compute IPA enrichment. DEGs that positively and negatively correlated with a canonical pathway were assigned a positive and negative activation z-score value respectively. The right-tailed Fisher's exact test was used to calculate a *P-val* determining the probability that each pathway assigned to the data set was due to chance alone, and *P-vals* were corrected for multiple comparisons (Benjamini-Hochberg). To display the non-redundant significantly enriched pathways in ***Figure 2c***, we selected for pathway terms that had at least 10 DEGs associated with it, passed the pathway enrichment significance cutoffs: *P-val* < 0.05 and FDR *Q-val* < 0.1, and finally was a significant pathway in at least 3 regions (***Figure 2c, Supplementary Table TS13***).

Bulk ChIP-seq data generation

For each ChIP-seq assay approximately 5-25 mg of frozen brain tissue per replicate per region was aliquoted and thawed on ice in 1 ml PBS buffer. Tissue was homogenized using a manual glass douncer with 7-15 slow strokes on ice. The cell suspension was filtered with a 50 µm cell strainer (Falcon) by spinning at 2000 rpm for 1 min at 4°C in a swing bucket centrifuge (Eppendorf Centrifuge 5810R). Pellets were then washed twice with cold PBS, crosslinked with 1% formaldehyde for 12 min at room temperature and excess formaldehyde quenched by addition of glycine (0.625 M). Cells were lysed with FA buffer and nuclei were collected and re-suspended in 300 µl SDS lysis buffer (1% SDS, 1% Triton X-100, 2 mM EDTA, 50 mM HEPES-KOH, pH 7.5, 0.1% Sodium deoxycholate, Roche 1X Complete protease inhibitor). Nuclei were lysed for 20 min, after which sonication was used to fragment chromatin to an average size of 200–500 bp (Bioruptor Next gen, Diagenode). Protein-DNA complexes were immuno-precipitated using 3 µg of H3K27ac antibody of the same lot no. for all 108 (27 regions x 2 replicates x 2 treatment groups) ChIP experiments (Active Motif, Cat# 39133) coupled to 50 µl protein G Dynabeads (Invitrogen) overnight. The beads were washed and protein-DNA complexes were eluted with 150 µl of elution buffer (1% SDS, 10 mM EDTA, 50 mM Tris-HCl, pH 8), followed by protease treatment and de-crosslinking at 65°C overnight. After phenol/chloroform extraction, DNA was purified by ethanol precipitation. 5% of sheared chromatin was aliquoted and treated with pronase and RNase following de-crosslinking in the same manner as the ChIP DNA. To prepare pooled input libraries for each brain region, DNA was quantified and equal amounts of FT and Sham samples were pooled together. 100 ng of pooled DNA from input and ChIP samples were used for library preparation. Library preparation was performed as described here [7]. After 15 cycles of PCR using indexing primers, libraries were size selected for 300-500 bp using AMPure beads for size selection. 4 ChIP libraries were pooled and sequenced in one lane of 76 bp paired-end type, using the Illumina HiSeq 2500 with V4 reagents. All input libraries were multiplexed and sequenced in one HiSeq lane.

ChIP-seq data analysis

a) Read alignment, peak calling and peak height normalization:

ChIP-sequencing reads were aligned to the Rn5 rat genome (Ensembl). Each brain region had 4 ChIP-seq libraries (2 replicates per region, 2 treatment groups – FT, Sham). DFilter was used to call peaks from each ChIPseq library. This peak list was further filtered to construct a consensus peak set using the following filtering cut-offs: DFilter *P-val* < 1e-6 in at least one out of four libraries and a minimum of 20 normalised read counts per 1kb window, in at least two out of the four libraries. This filtering yielded a consensus peak set ~48K peaks across 27 brain regions.

Normalisation: The number of reads in each library within 200bp bins were counted and scaled to normalise for sequencing depth. These binned counts were then normalised for GC bias, using the input (no antibody) ChIP-seq library. Following the above normalisations, within each peak region the sum of binned counts were defined as the peak height and blurred within a 1kb-wide mean filter. These computed peak heights for all peaks in the consensus set were collated as one matrix.

b) Visualisation using tSNE and PCA analysis:
Read counts of each brain region were quantile normalised, centred and visualised on PC1 and PC2 loadings of PCA. Read counts of Sham and FT samples within each brain region were also visualised by PCA (***Supplementary Figure S7a-b)***.

c) Differential peak calling (DA peaks):
We used DESeq2 to call differentially acetylated peaks (DA peaks) from the consensus peak set (~48K peaks across 27 brain regions). DA cut-offs were as follows: absolute $\log_2$FC: $\geq \log_2(1.25)$ and FDR *Q-val* ≤ 0.1. We called a peak in any region as a differential peak under the following conditions: a) the peak featured in the consensus list, b) the peak passed the DESeq2 DA cut-offs. This resulted in the identification of a 4,511 significant DA peak set, wherein any peak was DA in at least one brain region (***Figure 3a***, ***Supplementary Table TS10-11***). Next, as a quality metric we i) calculated the correlation between the different replicates per brain region and ii) compared the fraction of region-wise DA peaks to ChIP-seq signal quality in that respective region. ChIP-seq signal quality for every brain region was defined as the ratio of mean read count of top 10,000 peaks in that region to the read count of random genomic bins not lying on peaks (***Supplementary Figure S7c-f***).

Functional enrichment of DA peaks

GREAT enrichment analysis for co-regulated gene cluster modules and IPA enrichment tests for region-wise DA peaks,

We performed k-means clustering (no. of clusters=8) on the DA peaks set (4511 DA peaks). To functionally annotate the 8 DA k-means modules, we used the GREAT [8] tool to compute statistically significant enrichment for any gene categories. DA peaks were associated with genes using GREAT's 'basal+extension association rule'. To test enrichment for each k-means module, DA peaks in that module constituted the foreground set, while the union of the remaining modules served as the background set. Gene ontology categories with enrichment ratio ≥ 1.5 and FDR *Q-val* ≤ 0.05 were retained. Additionally, we discarded the enriched gene category if less than 5 genes were associated with DA peaks in that category. Redundancy between two functional categories was defined as overlap of $\geq 40\%$ in the number of genes

associated with DA. To display the non-redundant significantly enriched gene categories in ***Figure 3d***, we further selected the top non-redundant category terms from GREAT. The complete list of GREAT results are listed in ***Supplementary Table TS13a***.

IPA enrichment tests were performed on DA peak sets of the top 6 highly ranked regions (as per DA peak ranking). Region-wise upregulated and downregulated peaks were considered separately as foreground sets, while the consensus peak set formed the background set. IPA pathway gene sets that were significantly enriched in the DEG set of these 6 brain regions were selected (IPA analysis of RNA-seq datasets). DA peaks were annotated to a gene that was within 1Mbp of the peak. Fisher's exact test was used to calculate *P-val*s (***Figure 3c, Supplementary Table TS13b)***.

Cell type enrichment:

To test the enrichment of DA k-means modules for cell type specific markers, we used the BRETIGEA database as described in the RNA-seq methods above. DA peaks were annotated to the nearest gene, whose TSS fell within 1Mbp of the peak. For each cluster, enrichment of annotated genes in that cluster (against a background of all annotated genes) with cell type-specific genes from the BRETIGEA database was assessed through Fisher's exact test. This process was repeated for all cell types and brain regions, and then corrected for multiple testing on a cell type level (Benjamini-Hochberg) ***(Figure 3d, Supplementary Table TS14)***.

Motif Analysis:

The eight DA k-mean modules were tested for enrichment of TF-binding motifs. We used the HOMER pipeline's findMotifsGenome.pl script (http://homer.ucsd.edu/homer/ngs/peakMotifs.html). The position weight matrices (PWM) of motifs were drawn from the TRANSFAC vertebrate database (https://genexplain.com/transfac/). We listed the top 3 enriched, non-redundant motifs in ***Figure 3d*** (FDR *Q-val* < 0.01). The complete list is provided in ***Supplementary Table TS15***.

Comparison of fold changes in Histone acetylation and RNA-seq counts

In order to find coherence between changes in histone acetylation and RNA levels (***Supplementary Table TS12b)***, we specifically looked at the differential H3K27ac level at promoters of DEGs. For this purpose, we first estimated the read count of H3K27ac ChIP-seq within 1 kb of transcription start site of every gene. The read count of promoters from replicates in every brain region was normalised using quantile-quantile normalization before calculating fold changes between fluoxetine and sham samples. We then calculated the spearman correlation of this log2 (fold-changes) for DA peaks at promoters and compared it with the corresponding log2 (fold-changes) of DEGs (as called by DEseq2). DEG cut-offs were

as follows: absolute log2FC ≥ log2 (1. 5) and FDR Q-val ≤ 0.1, DA promoter cutoffs (ratio of normalised read-counts) for this analysis was: absolute log2FC ≥ log2 (1. 5); no FDR cutoff was applied.

Rank-rank hypergeometric overlap analysis (*Figure 4a*)

Overlaps between the differential expression of the two independent RNA-seq comparisons were visualized and measured with a rank-rank hypergeometric overlap analysis. dorDG and venDG bulk RNA-seq datasets were compared by creating a threshold-free list of DEGs that were ranked by increasing log fold-change. We then used the RRHO2 "stratified" method to detect the overlap between genes differentially expressed in the same or opposite directions, where the bottom left and top right quadrant display overlap of genes with concordant differential expression, and the top left and bottom right discordant overlap.

Single cell RNA-seq data generation

dorDG and venDG tissues were punched from fresh brains of the two treatment groups. Single cell suspension from one portion was prepared using the papain dissociation system (Worthington, Cat# LK003150) as per manufacturer's instructions. Single nucleus suspension from an aliquot was prepared according to the demonstrated protocol for nuclei isolation from mouse brain tissue by 10x Genomics. Following manufacturer's recommendations for the 10x Genomics Chromium single cell 3' reagent kit v2, single cell and single nucleus suspensions were diluted to a range of between 1,200 and 1,300 cells or nuclei per µl and loaded into each well of the 10x Genomics single cell chip A, targeting a recovery of 4000 cells and nuclei per reaction. 12 to 13 cycles were used for cDNA amplification. Libraries were prepared and each library was sequenced on the Illumina HiSeq 4000 with 26 cycles for read 1, 100 cycles for read 2, and 8 cycles for i7 index.

Filtered matrices from Cell Ranger were analyzed using Seurat v3.2 (https://github.com/satijalab/seurat) in R v3.6.1. We used Reference Component Analysis (RCA2)[118], to divide cells into seven major cell types: (i) neurons, (ii) astrocytes, (iii) mature oligodendrocytes, (iv) OPCs, (v) myelinating oligodendrocytes, (vi) endothelial and (vii) microglia. To do so, we projected cells against RCA's in-built mouse brain panel and assigned each cell to the cell type that showed the highest Pearson correlation. We verified these assignments (i) by considering the expression of canonical marker genes and by (ii) calling differentially expressed genes (DEGs) for each major cell type cluster using Seurat's *FindAllMarkers* function with default parameters. Using RCA2's cell type specific quality control functionality, we applied stringent cut-offs for all seven major cell types separately to discard cells of low

quality while preserving cell type. Libraries with number of detected genes (NODG) < 1000 genes, UMIs > 20,000 (unique molecular identifiers) and proportion of mitochondrial reads > 25%, were filtered from the dataset. The Seurat integration method based on identification of "anchors" between pairs of datasets was used to eliminate biological and technical batch effects of the samples and scRNA-seq libraries. Subclustering and further downstream analysis was then done using the Seurat pipeline. Dimensional reduction was carried out using the first 30 principal components, with clusters identified using K-nearest neighbours at a resolution of 0.8. Specific cluster markers were identified using the built-in Seurat function *FindAllMarkers*, with a minimum $\log_2$ fold-change of 0.25 and expression in > 25% of cells. Clusters were annotated using common marker genes from the literature and previously published datasets. We identified 13 cell types, out of which cluster 'Endo2' that resembled Hba+ cells and was therefore removed from further analysis. We finally arrived at 12 cell types in dorDG and venDG single cells (Data QC: ***Supplementary Figure S8a-g***, ***Supplementary Table TS16)***. The cell type clusters and the cluster-specific markers are listed in ***Supplementary Table TS17, Supplementary Figure S9***. Cell type proportions statistics shown in ***Figure 4c*** are provided as a table in ***Supplementary Table TS18.***

Single cell DEG calling:

To identify DEGs within each cell type, we first created pseudo-bulk expression vectors for each replicate using Seurat, giving us 5 replicates for both Sham and FT groups. To remove outlier replicates, we first discarded genes with an average expression value (across both groups) of less than 0.5 transcripts per 10k (tp10k < 0.5). Outliers were then removed using the following procedure: within each group (Sham or FT), we 1) scaled the pseudo-bulk vectors across genes, 2) calculated the pairwise Euclidean distance between the scaled replicates, 3) for each replicate, took the median of the pairwise distances (MPD), 4) calculated the median absolute deviation (MAD) using the median of the pairwise distances across replicates, and 5) removed replicates that were at least 3 MADs away from the median of the MPDs.

Following outlier removal, we again discarded genes with a tp10k < 0.5, after which we transformed the expression vectors using $\log_2$ (expression+0.01), where 0.01 denotes the pseudocount. We then called DEGs using Student's t-test on the transformed values, after which we corrected t-test p-values for multiple testing. To calculate $\log_2$FC values, we applied the pseudocount to all values, and then took the $\log_2$ of the mean of FT values divided by the mean of Sham values. Genes with absolute ($\log_2$FC) $\geq$ $\log_2$(1.25) and an FDR $Q\text{-}val \leq 0.2$ were considered DEGs (***Figure 4d***, ***Supplementary Table TS19)***. Functional enrichment of DEGs were tested using GSEA-Go enrichment function-'enrichGO' (***Supplementary Table TS20*)**.

Canonical Oxphos signalling module score:

We computed a gene module score in single-cell space using Seurat's *AddModuleScore* implementation with default parameters. A module is a list of genes that defines a phenotype and in our case, we defined the 'oxphos' module as a list of 150 genes from the 'oxidative phosphorylation pathway' curated in the Ingenuity Knowledge base (IPA). The module scores for each cell type and each group (FT and Sham) were computed separately and the differences were assessed using a Wilcoxon rank sum test, and the *P-val*s were corrected for multiple testing (***Figure 4f, Supplementary Table TS21***).

Validation of Mitochondrial and Oxphos trends:

a) Cell culture

C6 rat glioma cell line (ATCC CRL-2199, RRID:CVCL_5657) was cultured in DMEM (4.5 g/L glucose) (Gibco, Cat# 11995065) supplemented with 10% heat-inactivated fetal bovine serum (Gibco, Cat# 10082147) and incubated at 37°C with 5% CO_2 in a humidified atmosphere.

b) Drug treatment

C6 rat glioma cells were seeded at a density of 50,000 cells into T25 flasks and treated with fluoxetine (Sigma, Cat# F132) after overnight incubation at 37°C with 5% CO_2 in a humidified atmosphere. 6 h after addition of fluoxetine, either corticosterone (Sigma, Cat# 27840) or ethanol was added for the respective treatment groups and incubated for another 66 h (total 72 h).

c) Mito stress assay

After 72 h drug treatment, cells in all treatment groups were harvested and seeded at a density of 30,000 cells (in respective treatment media) into each well of a Seahorse XF96 V3 PS microplate (Agilent Technologies, Cat# 101085-004) for the mito stress assay and a 96-well black, clear bottom plate (Corning, Cat# 3904) for cell number normalisation. Cells were then left in the culture hood at room temperature for 1 h to prevent edge growth effects before incubation at 37°C with 5% CO_2 in a humidified atmosphere. After overnight incubation, the basal and maximal respiration/oxygen consumption rate (OCR) of cells in the Seahorse microplate were measured using the Seahorse XFe96 analyzer and mito stress test kit (Agilent Technologies, Cat# 103015-100) as per manufacturer's recommendations. Briefly, cells were washed twice with Seahorse XF DMEM medium, pH 7.4 supplemented with 10 mM XF glucose, 1 mM XF pyruvate, and 2 mM XF glutamine (Agilent Technologies). Following incubation at 37°C in a non-CO_2 incubator for 1 hour prior to the assay, 1.5 µM of oligomycin, 1 µM of carbonyl cyanide-4-(trifluoromethoxy) phenylhydrazone (FCCP), and 0.5 µM of a mixture of rotenone and antimycin A were sequentially injected during the assay. Cells in the 96-well black, clear bottom plate were stained with Hoechst 33342 (Sigma, Cat# 62249) and the fluorescence signal was measured with the GloMax plate reader (Promega). OCR

values from the mito stress assay were normalized to cell number using the fluorescence signal (***Figure 4h***).

d) MitoTracker Red and Cytochrome c Immunofluorescence; image acquisition and analysis

Cells were plated in clear 12-well plates overnight at 50-80% confluency. After attachment of cells to the plate, MitoTracker Red CMXRos (300 nM, Thermo Fisher Scientific) was added into live cells for 20 min at 37°C. Cells were then washed three times for 5 min in PBS and fixed in 4% PFA for 15 min at room temperature. Following 3 washes of PBS, cells were then incubated in blocking buffer (5% horse serum, 1% BSA, 0.2% Triton-X in PBS) for 1 h at room temperature. Cytochrome c antibody (BD Pharmigen, clone 6H2.B4) was added at 1:1000 dilution in blocking buffer and incubated overnight at 4°C. The next day, cells were washed three times for 5 min in PBS and incubated with Alexa Fluor 488-conjugated anti-mouse antibody (Life Technologies) at 1:400 dilution in blocking buffer for 1 h at room temperature. Nuclei were stained with DAPI in blocking buffer.

Imaging was performed with a Zeiss Axio Observer Z1 epifluorescence microscope. Images were then analysed using a custom MATLAB script, with analysis being performed blind to the treatment conditions. For each image, we determined the mean background fluorescence for every channel by drawing regions of interest (ROIs) across the image where there were no cells, and then computing the average pixel intensity across all background ROIs. We then drew, for each cell, an ROI around its border as defined by the MitoTracker Red staining. We then computed the average pixel intensity per cell, defined as the average intensity within the cellular ROI minus the mean background intensity value, for each channel. Similarly, total intensity per cell was calculated by multiplying the average pixel intensity by the number of pixels in the cellular ROI. Intensity values for each channel and cell were then normalized to the average value of cells in the vehicle (ethanol-treatment) condition. We used a one-way ANOVA with multiple comparisons to compare the normalized values across the 4 conditions (ethanol, corticosterone, corticosterone+fluoxetine, fluoxetine). Statistical analysis and graphs were generated using Prism 8 (Graphpad Holdings, LLC) (***Figure 4g***).

SCENIC analysis:

To identify upstream regulators and their downstream targets (regulons), we implemented the SCENIC pipeline [9] with several modifications. For each region, we first removed single cells from the total matrix if they belonged to outlier replicates (see above on single cell DEG calling). This method removed ~5% of cells and did not have a sizeable impact on cell numbers. We then transformed the raw counts matrix into a $\log_2$(counts+1) matrix, where 1 denotes the pseudocount, and passed that into SCENIC to calculate correlations. To calculate links between TFs and their downstream targets, we used Grnboost2

from the python implementation of SCENIC, pySCENIC, rather than GENIE3 from the R implementation, as this greatly sped up calculations. The link file that resulted (also known as adjacencies in pySCENIC) was then passed back into SCENIC for construction of regulons and AUC scoring. To construct regulons, we used the database:

'mm10__refseq-r80__500bp_up_and_100bp_down_tss.mc9nr.feather'.

Following regulon identification and AUC scoring, we calculated cell type specific markers and differentially activated regulons (DARs). For each cell type, potential markers needed to have nonzero AUC values in at least 10% of cells in that cell type. We then calculated marker enrichment (or $\log_2$ fold-change) by taking the $\log_2$ of the mean of the AUC values in that cell type divided by the mean of the AUC values in all other cell types. P-values were calculated using Wilcoxon, again comparing the cell type versus all other cell types, and then adjusted using FDR (***Supplementary Table TS22a)***.

Likewise, to calculate DARs, potential DARs needed to have nonzero AUC values in at least 2% of cells in either the Sham or FT group. We then calculated marker enrichment (or $\log_2$ fold-change) by taking the $\log_2$ of the mean of the AUC values in the FT group divided by the mean of the AUC values in the Sham group. P-values were calculated using Wilcoxon applied to the two groups and adjusted using FDR (***Figure 5a-b***, ***Supplementary Figure S10***, ***Supplementary Table TS22b)***.

NATMI Signalling analysis:

To infer differential signalling interactions between FT and Sham, we first used NATMI's [10] (git commit: f35f677) `ExtractEdges.py` command to compute expression and specificity scores for each interaction edge, defined by a sender cell type, receiver cell type, ligand and receptor. Scores were computed using the counts-per-10k values extracted from the scRNA-seq expression within each sample. Then, for each interaction, we tested the difference in the expression and specificity scores using the Wilcoxon-rank sum test. We reasoned that top differential interactions should be relatively specific, and that the ligands and receptors should be expressed by a non-negligible fraction of cells within the sender and receiver cell types in the condition with the higher score. Hence, to prioritize top differential interactions for inspection, we applied the following filters: absolute difference in specificity score > 0.03; specificity score in condition with higher score > 0.05; average fraction of ligand expressed in sender cell type in samples from the condition with the higher score > 0.1; average fraction of receptor expressed in receiver cell type in samples from the condition with the higher score > 0.1; unadjusted Wilcoxon p-value testing difference in expression or specificity score < 0.05. This resulted in 22 top interactions for dorDG and 6 top interactions for venDG. Then, for each unique ligand-receptor pair, the $\log_2$ fold-change in average expression score (calculated using a pseudocount of 1) and the average difference in specificity score between the conditions

were visualized as a dotplot, with dot size corresponding to the unadjusted Wilcoxon p-value (***Figure 5c-d***, ***Supplementary Figure S11, Supplementary Table TS23)***.

References in Methods:

1. Paxinos G, Watson C. The Rat Brain in Stereotaxic Coordinates. 1982: iv.

2. Biernacka JM, Sangkuhl K, Jenkins G, Whaley RM, Barman P, Batzler A, et al. The International SSRI Pharmacogenomics Consortium (ISPC): a genome-wide association study of antidepressant treatment response. Transl Psychiat. 2015;5:e553.

3. Uher R, Perroud N, Ng MYM, Hauser J, Henigsberg N, Maier W, et al. Genome-Wide Pharmacogenetics of Antidepressant Response in the GENDEP Project. Am J Psychiat. 2010;167:555–564.

4. Howard DM, Adams MJ, Clarke T-K, Hafferty JD, Gibson J, Shirali M, et al. Genome-wide meta-analysis of depression identifies 102 independent variants and highlights the importance of the prefrontal brain regions. Nat Neurosci. 2019;22:343–352.

5. Lambert J-C, Grenier-Boley B, Chouraki V, Heath S, Zelenika D, Fievet N, et al. Implication of the Immune System in Alzheimer's Disease: Evidence from Genome-Wide Pathway Analysis. J Alzheimer's Dis. 2010;20:1107–1118.

6. Kranzler HR, Zhou H, Kember RL, Smith RV, Justice AC, Damrauer S, et al. Genome-wide association study of alcohol consumption and use disorder in 274,424 individuals from multiple populations. Nat Commun. 2019;10:1499.

7. Sun W, Poschmann J, Rosario RC-H del, Parikshak NN, Hajan HS, Kumar V, et al. Histone Acetylome-wide Association Study of Autism Spectrum Disorder. Cell. 2016;167:1385-1397.e11.

8. McLean CY, Bristor D, Hiller M, Clarke SL, Schaar BT, Lowe CB, et al. GREAT improves functional interpretation of cis-regulatory regions. Nat Biotechnol. 2010;28:495–501.

9. Sande BV de, Flerin C, Davie K, Waegeneer MD, Hulselmans G, Aibar S, et al. A scalable SCENIC workflow for single-cell gene regulatory network analysis. Nat Protoc. 2020;15:2247–2276.

10. Hou R, Denisenko E, Ong HT, Ramilowski JA, Forrest ARR. Predicting cell-to-cell communication networks using NATMI. Nat Commun. 2020;11:5011.
