# Supplementary material for: Integrative multi-omics landscape of fluoxetine action across 27 brain regions reveals global increase in energy metabolism and region-specific chromatin remodelling
Source: Mol Psychiatry. 2022 Sep 2;27(11):4510–25. doi: 10.1038/s41380-022-01725-1 (PMC9734063; doi:10.1038/s41380-022-01725-1)
Supplement: Supplementary file 2 — Supplementary Figures [file 41380_2022_1725_MOESM2_ESM.pdf]

<u>**Supplementary Figures**</u>

Integrative multi-omics landscape of fluoxetine action across 27 brain regions reveals global increases in energy metabolism and region-specific chromatin remodeling

Supplementary Fig. S1a

tSNE plot: average counts in all 27 regions

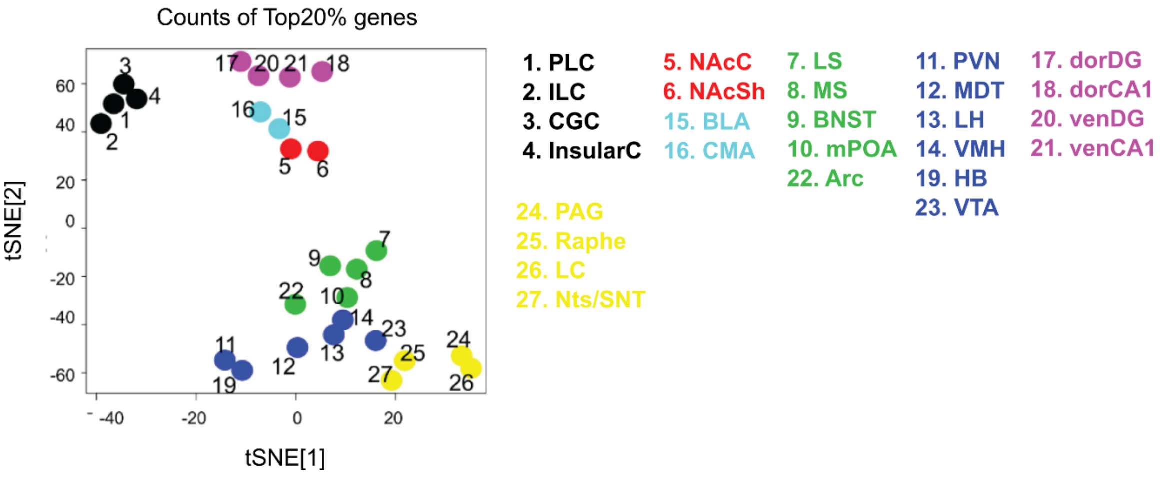

Supplementary Fig. S1

a. T-distributed stochastic neighbour embedding (tSNE) visualization of all samples per region: Top 20% of genes (average read counts of all samples) detected in each brain region were visualised using tSNE. Each dot represents a brain region, and the color of the dot denotes anatomically proximal region-groups.

Supplementary Fig. S1b

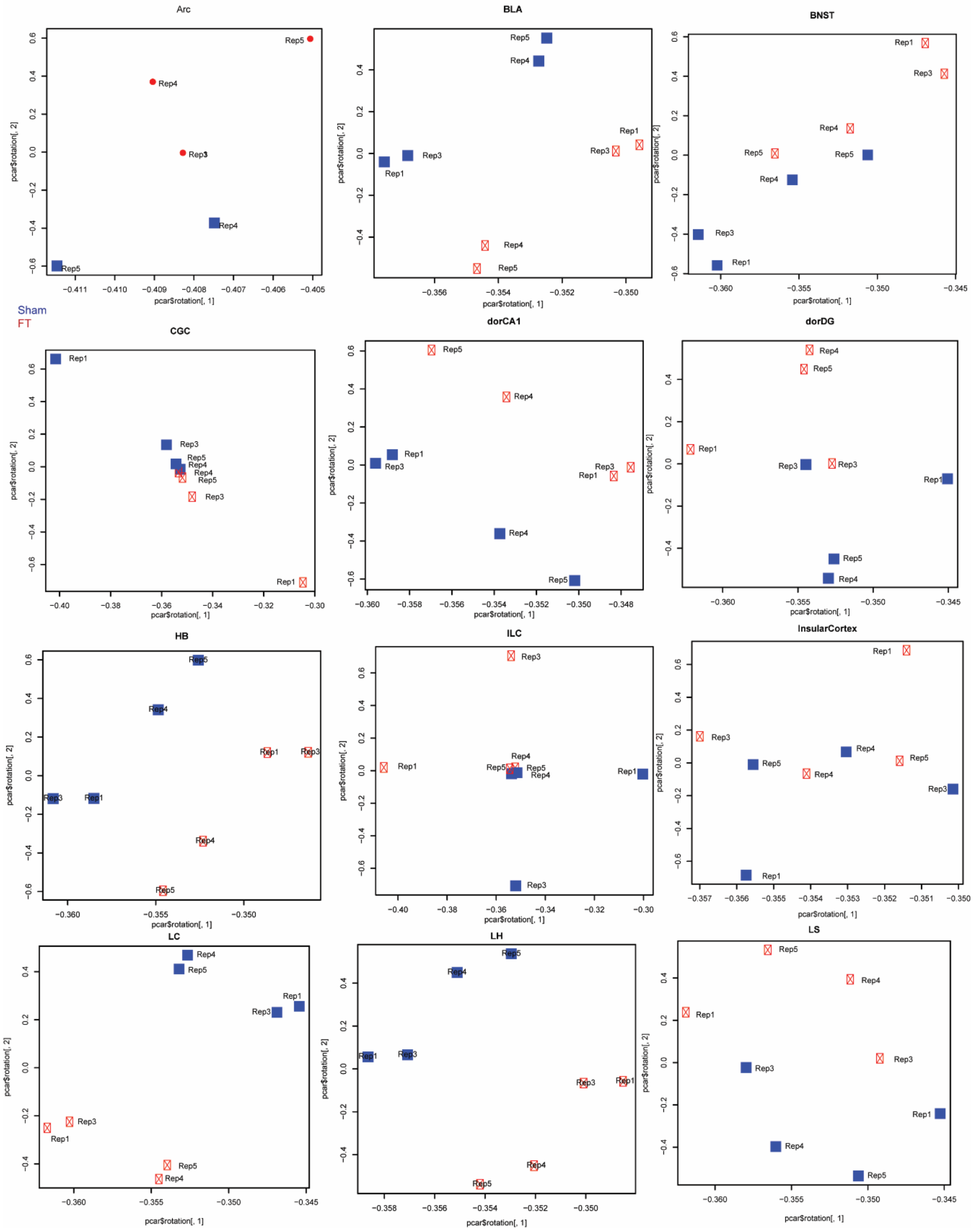

Supplementary Fig. S1

b. Principal Component Analysis (PCA) visualization of the Sham and FT replicates (Rep) per region: Normalised and batch corrected read counts of DEGs, per replicate per region were visualised on PC1 and PC2 loadings (plots for 27 brain regions are provided). Sham replicates are coloured blue and FT in red.

Supplementary Fig. S1b

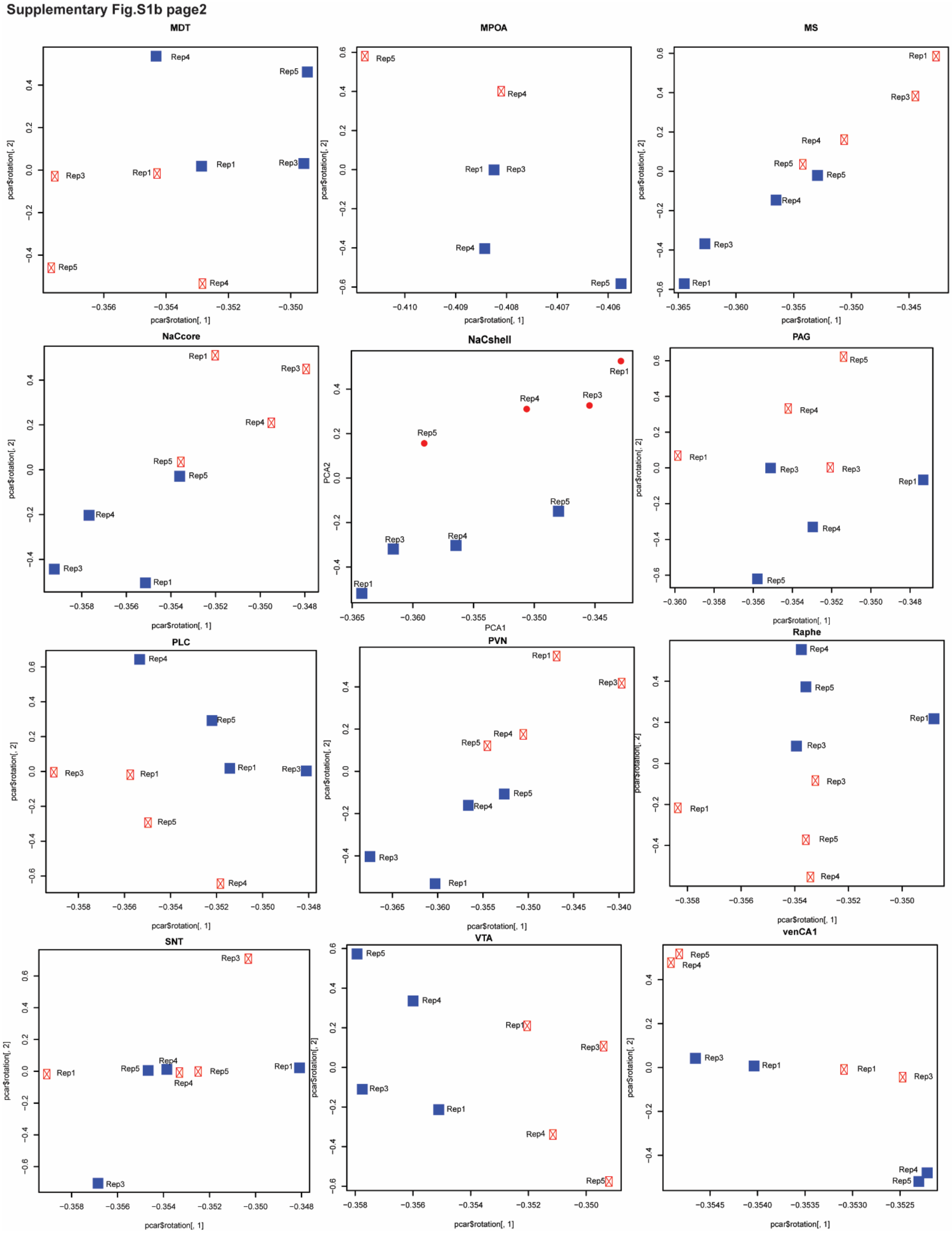

Supplementary Fig. S1b continued

Supplementary Fig. S2

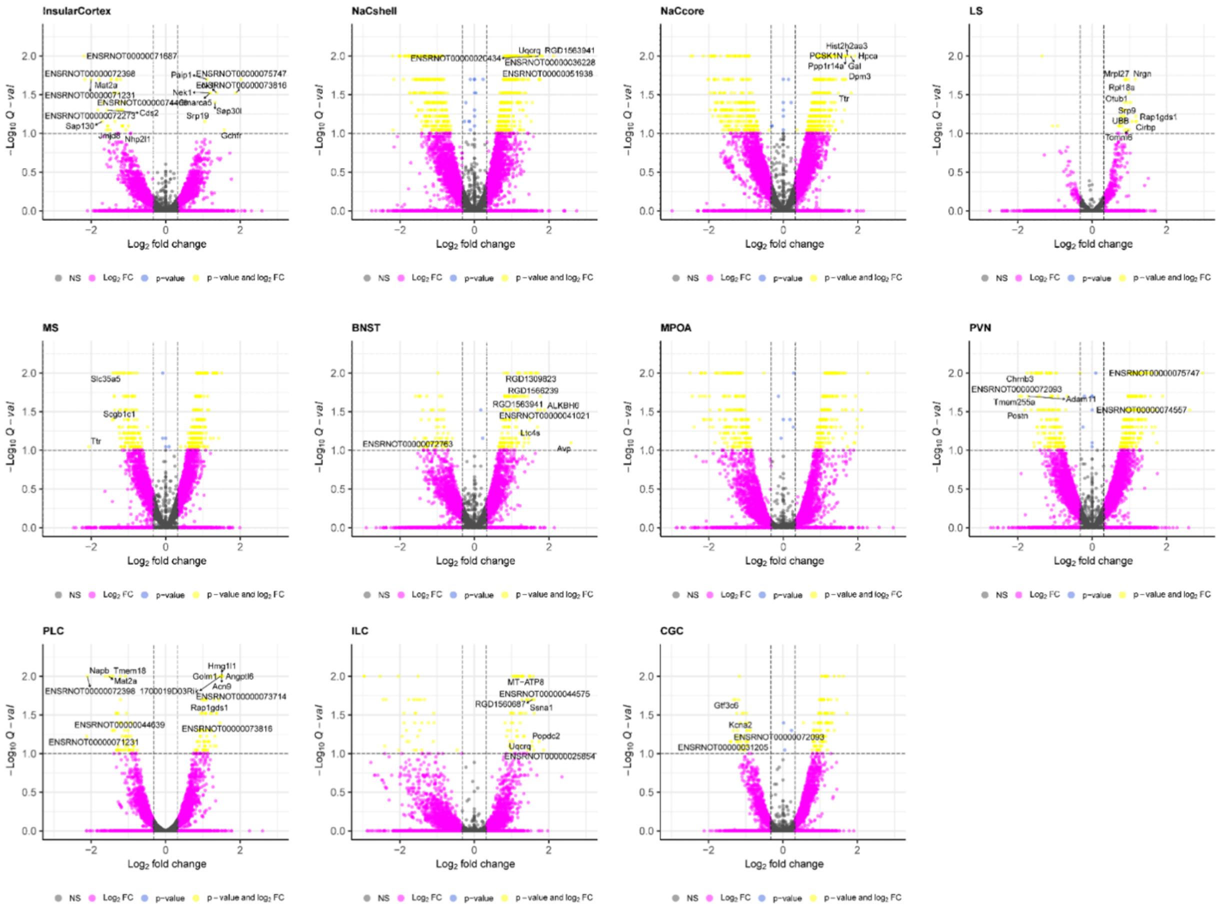

Supplementary Fig. S2

Volcano plots of $\log_2$-fold-change values (x-axis) and $-\log_{10}(Q\text{-}val)$ (y-axis) across the 27 brain regions. DEGs are marked in yellow (selected genes are labelled); genes with significant $\log_2$-fold-changes but not significant *Q-vals* are marked in magenta; the rest in grey.

Supplementary Fig. S2

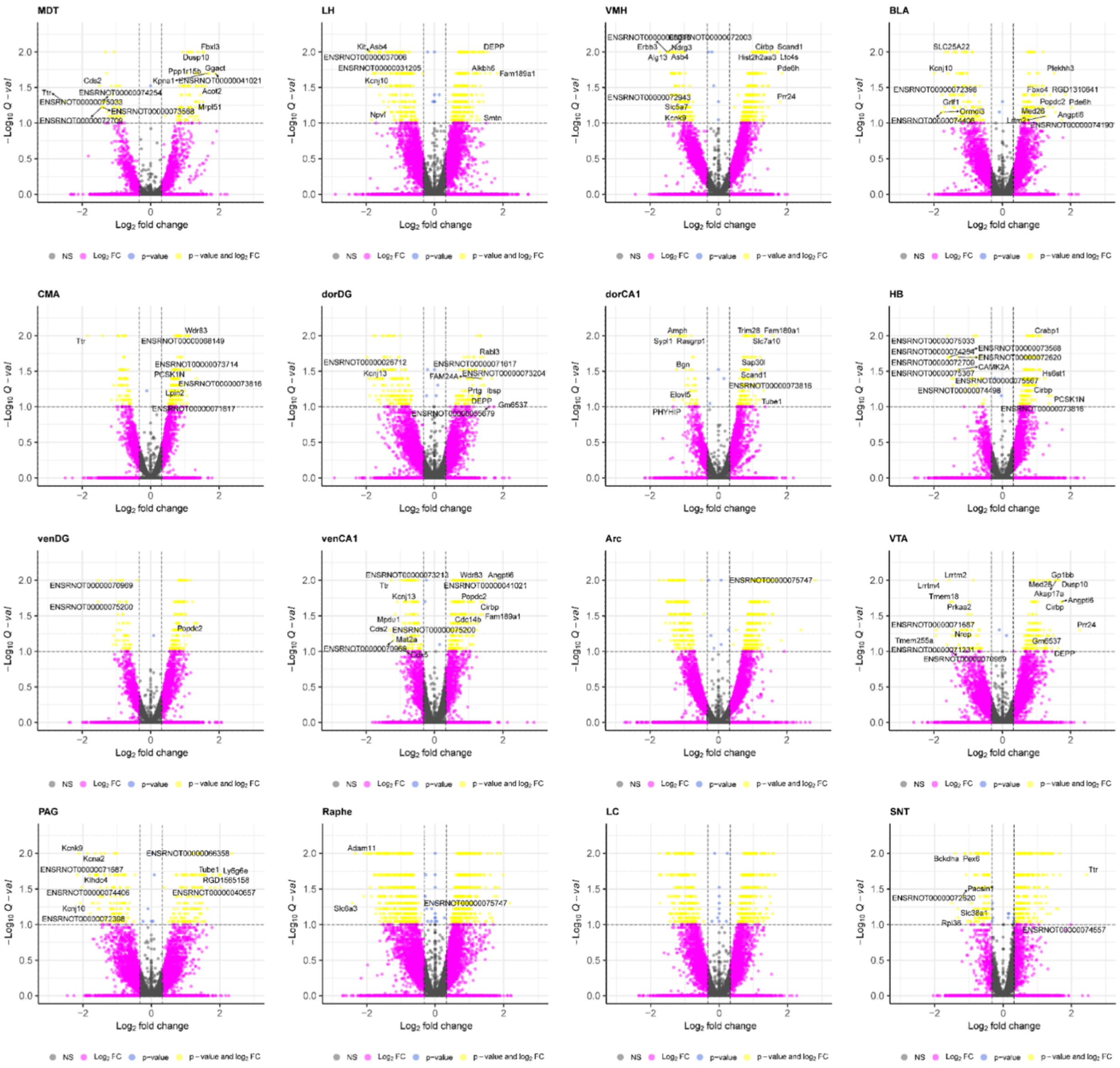

Supplementary Fig. S2 (continued)

Supplementary Fig. S3

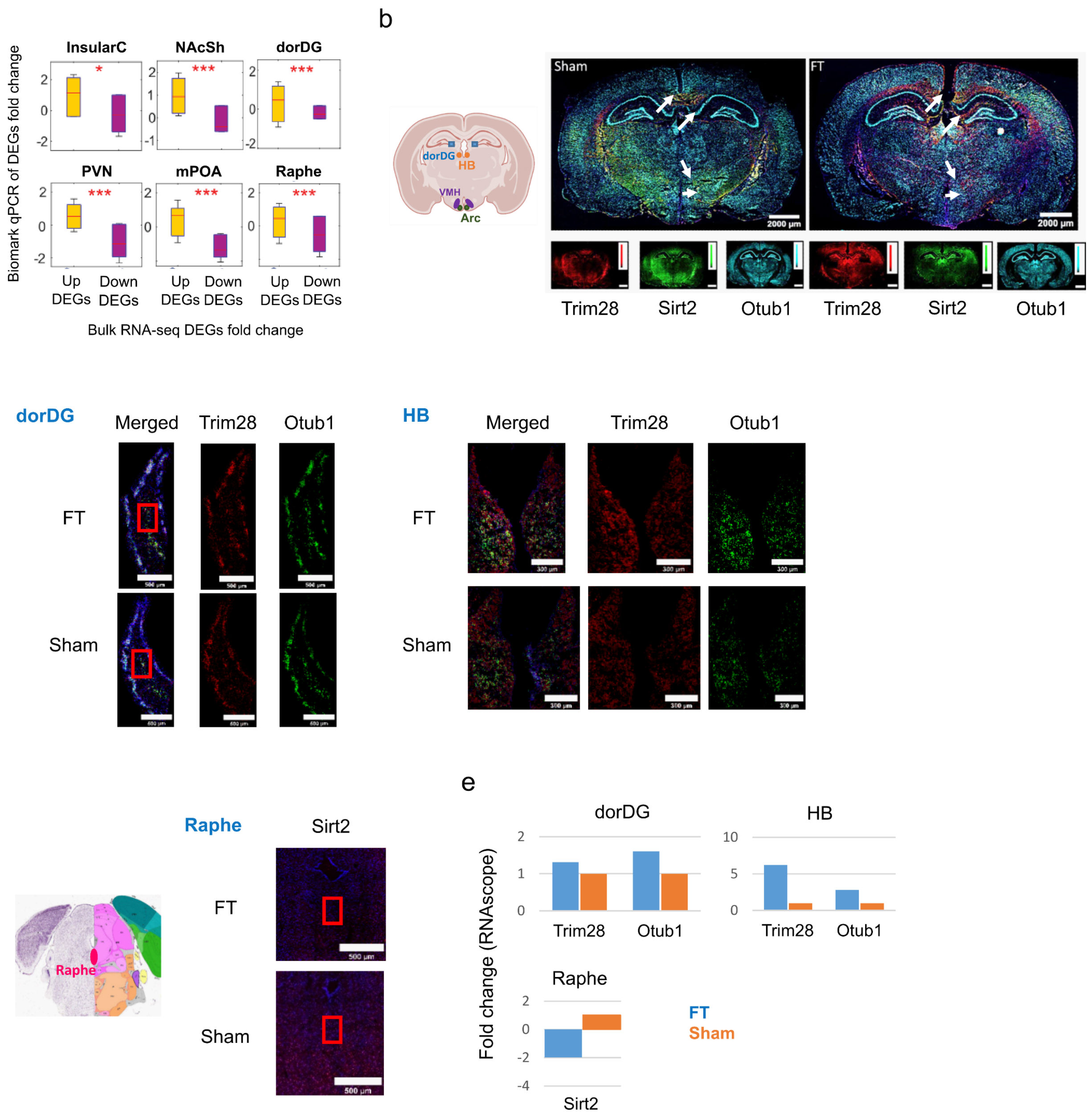

Supplementary Fig. S3

- **a.** High-throughput Biomark qPCR log_2FC values of 8 upregulated and 8 downregulated DEGs are compared to their corresponding RNA-seq DEG status in each of the listed six brain regions.
- **b.** RNAscope of 3 DEGs (Trim28, Otub1, Sirt2) in specific regions are shown. Scale bar indicates 200 µm.
- **c.** RNAscope images (zoomed-in) of the dorDG and Habenula (HB) regions are shown (DEGs: Trim28, Otub1).
- **d.** RNAscope images (zoomed-in) of the Raphe are shown (DEG: Sirt2).
- **e.** Bar plots showing the single molecule RNA counts in FT and Sham sections of 3 brain regions (dorDG, HB and Raphe).

Supplementary Fig. S4

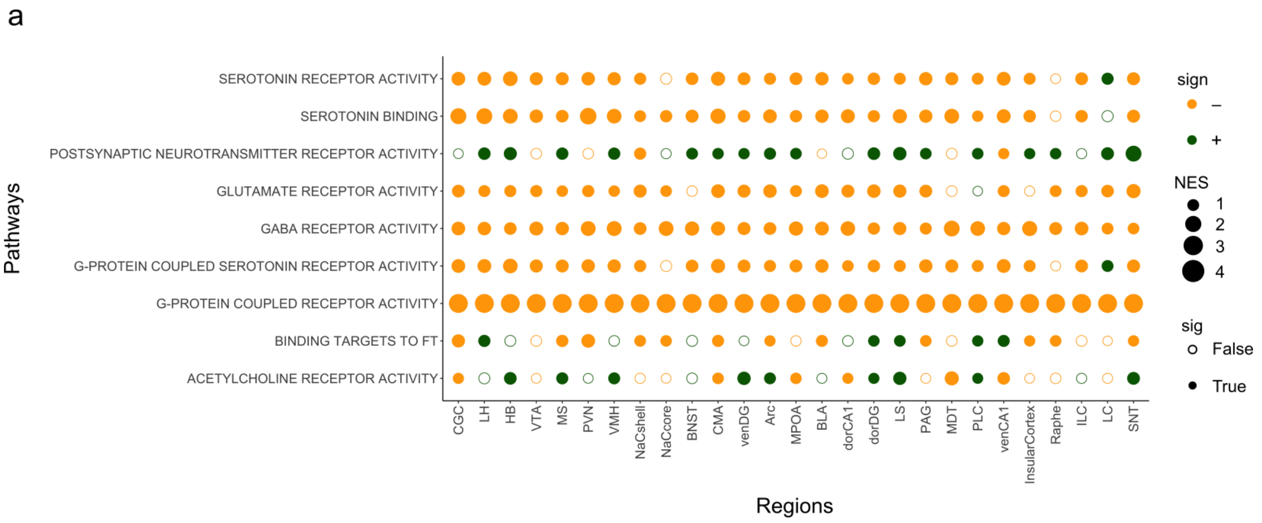

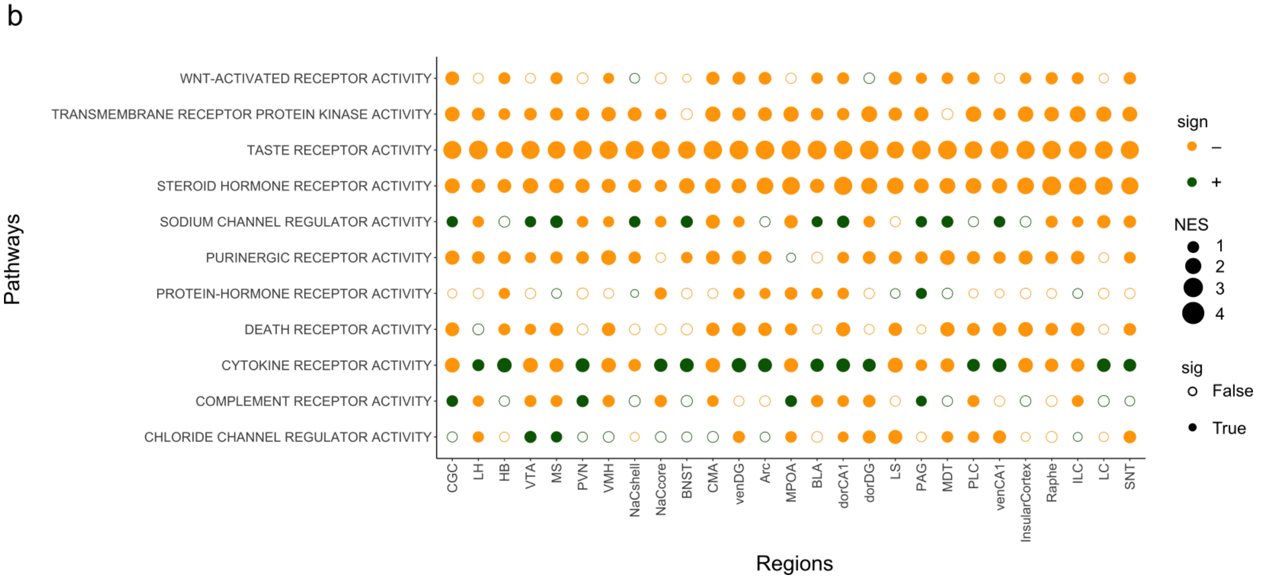

Supplementary Fig. S4

a. GSEA analysis of the effects of chronic fluoxetine on neurotransmitter receptor GO terms. GSEA normalized enrichment scores (NES) for GO terms (listed on the Y-axis). Upregulated sets are shown in green, downregulated in yellow. Closed circle indicates FDR *Q-val* <0.25, open circle denotes a non-significant FDR.

b. As in **a.**, but for non-neurotransmitter receptor GO terms.

Supplementary Fig. S5

a

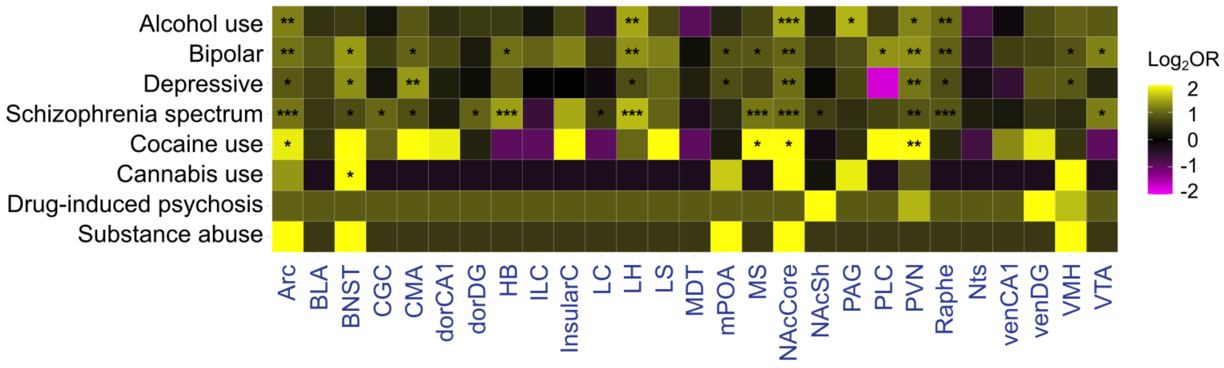

b

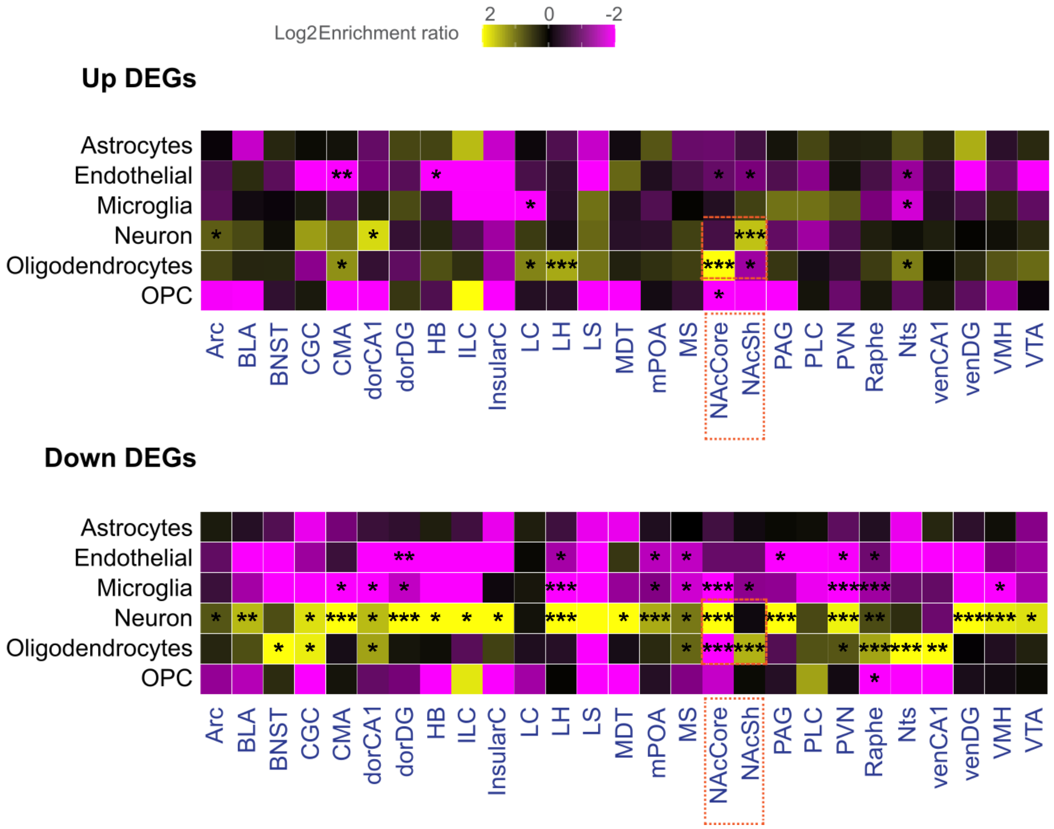

Supplementary Fig. S5

- **a.** PsyGeNET results (enrichment for genes associated with Psychiatric disorders). Log_2 enrichment ratios (Log_2OR) of region-specific DEGs with each set of psychiatric disorder-related genes from the PsyGeNET database. *** indicates *P-val* <1e-3, ** *P-val* <1e-2, * *P-val* <1e-1.
- **b.** RNA cell type enrichment. Log_2 enrichment ratios of upregulated (*top*) and downregulated (*bottom*) cell type-specific DEGs with their corresponding cell type-specific genes in the 6 major brain cell types per the BRETIGEA database. *** indicates *P-val* <1e-3, ** *P-val* <1e-2, * *P-val* <1e-1.

Supplementary Fig. S6

a

Correlation of Fluoxetine response (this study, rats) to Fluoxetine responders (Res) and non-responders (nonRes) in CORT-stressed mice (Carazo-Arias et al., 2022)

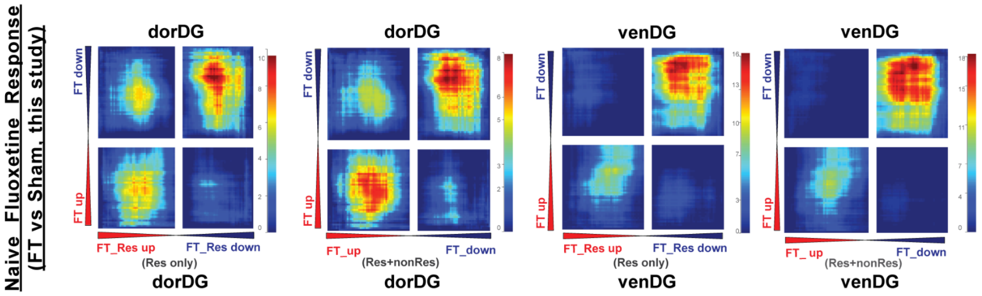

Fluoxetine Response in Stressed mice (CORT):
(FT Res & nonRes + Stressed) vs (Saline+Stressed), Carazo-Arias et al., 2022

b

Correlation of Fluoxetine response (this study, rats) to Imipramine responders in stress susceptible mice (Bagot et al., 2017)

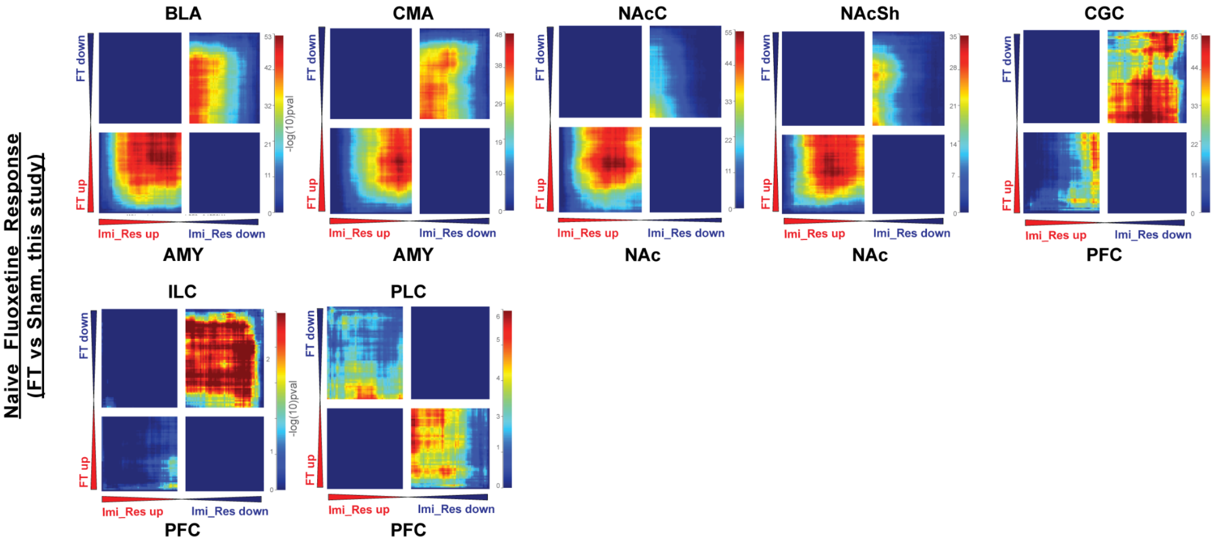

Imipramine Response in Stress Susceptible mice (Chronic social defeat model):
(Imi responders on Stressed) vs (Saline+Stressed), Bagot et al., 2017

c

Correlation of Fluoxetine response (this study, rats) to Fluoxetine responders in CV stressed mice (Anacker et al. unpublished)

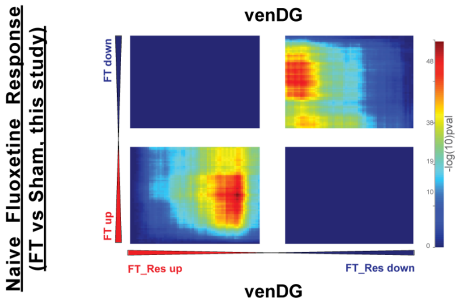

Fluoxetine Response in Chronic variable stressed mice:
(FT+Stressed) vs (Saline+Stressed)

Supplementary Fig. S6

a-c. Rank-rank hypergeometric overlap (RRHO) maps show the degree of differential expression (DDE; -$\log_{10}$(*p-value*)*direction of change) comparison between this study and prior literature and unpublished data. Pixels represent the overlap between the transcriptome of each comparison, with the significance of overlap (–$\log_{10}$(*P-val*) of a hypergeometric test) colour coded. Genes along each axis are sorted from most positive DDE (lower left) to most negative DDE (top right).

a. RRHO plots showing overlap in Fluoxetine response (this study, rats) with gene expression changes in Fluoxetine responders (Res) and non-responders (nonRes) in CORT-stressed mice [66]. Shown here are plots for two brain regions – dorDG and venDG. Differential analysis for identifying fluoxetine response was done as follows in the respective studies: FT vs Sham; Responders to FT in the CORT+FT group vs CORT control; Responders and Non-Responders to FT in the CORT+FT group vs CORT control.

b. RRHO plots showing overlap in Fluoxetine response (this study, rats) with gene expression changes in Imipramine responders in stress susceptible mice [65]. Shown here are plots for 7 brain regions featured in our study (BLA, CMA, NAcC, NAcSh, PLC, CGC and ILC), corresponding to 3 regions (Amygdala (AMY), NAc (nucleus accumbens) and PFC (prefrontal cortex)) in [65]. Differential analysis for identifying fluoxetine response was done as follows in the respective studies: FT vs Sham; Responders in the Chronic social defeat + Imipramine group vs Chronic social defeat + Saline group (control).

c. Correlation of Fluoxetine response (this study, rats) to Fluoxetine responders in mice exposed to chronic variable stress (CVS, Anacker et al. unpublished). Shown here are plots for venDG. Differential analysis for identifying fluoxetine response was done as follows in the respective studies: FT vs Sham; FT responders + CVS vs CVS control.

Supplementary Fig. S7

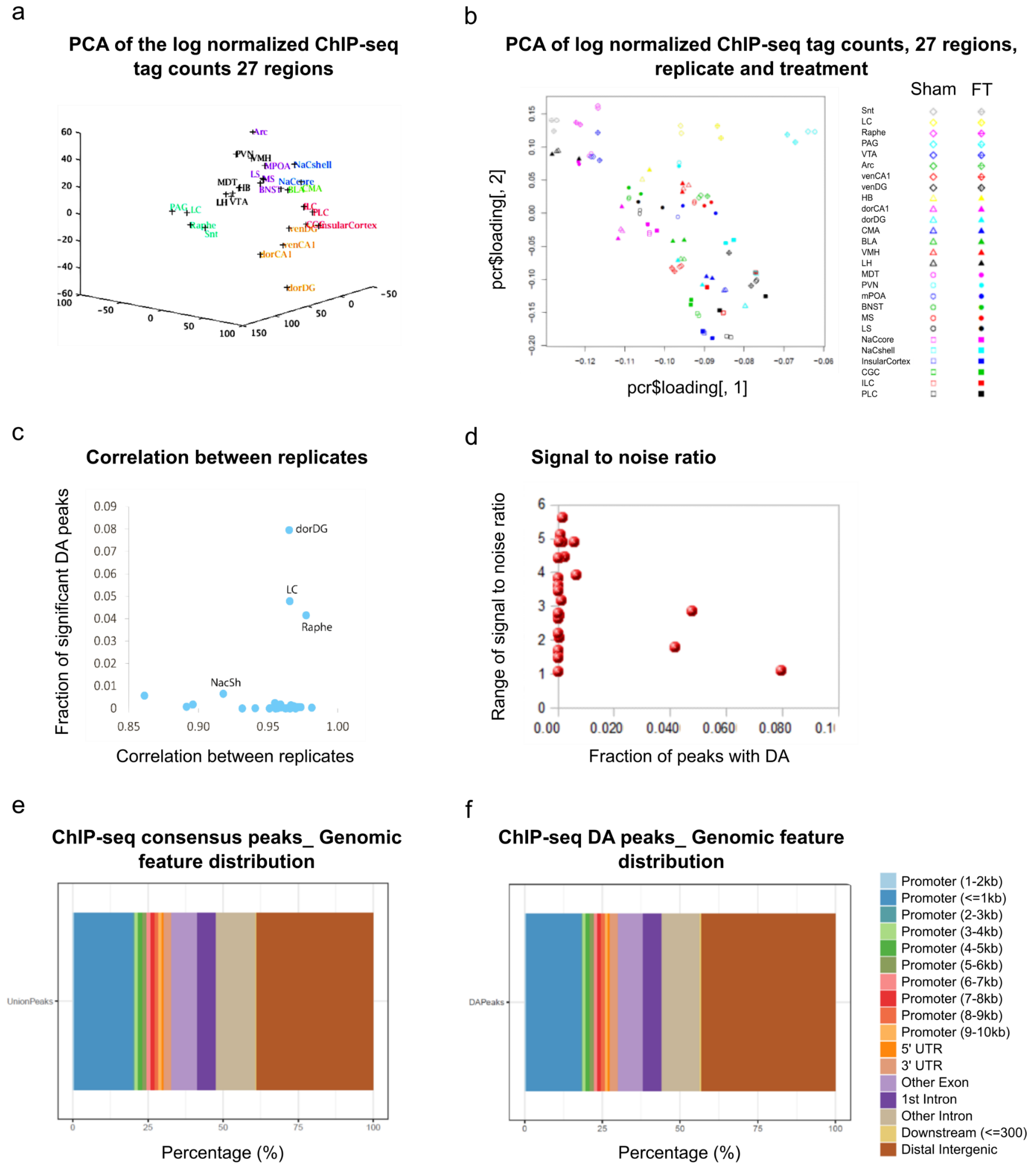

Supplementary Fig. S7

a. PCA of log-normalized ChIP-seq peak heights (all 27 regions are plotted). For every peak the average read-count of all Sham replicates were taken.

b. PCA of log-normalized ChIP-seq tag counts, 27 regions, replicate and treatment wise. Sham and FT libraries are represented by open and closed shapes respectively.

- **c.** Correlation between ChIP-seq replicate libraries versus the fraction of DA peaks. Each dot denotes a brain region.
- **d.** Signal-to-noise ratio versus the fraction of DA peaks. ChIP-seq signal quality was the ratio of the mean read count from the top 10,000 peaks to the mean read count of random genomic bins not lying on peaks. Each dot represents a brain region.
- **e.** Bar chart of the genomic distribution of peaks (consensus peak set) within introns/intergenic regions, exons, promoters and untranslated regions.
- **f.** Bar chart of the genomic distribution of peaks (DA peak set) within introns/intergenic regions, exons, promoters, and untranslated regions.

Supplementary Fig. S8

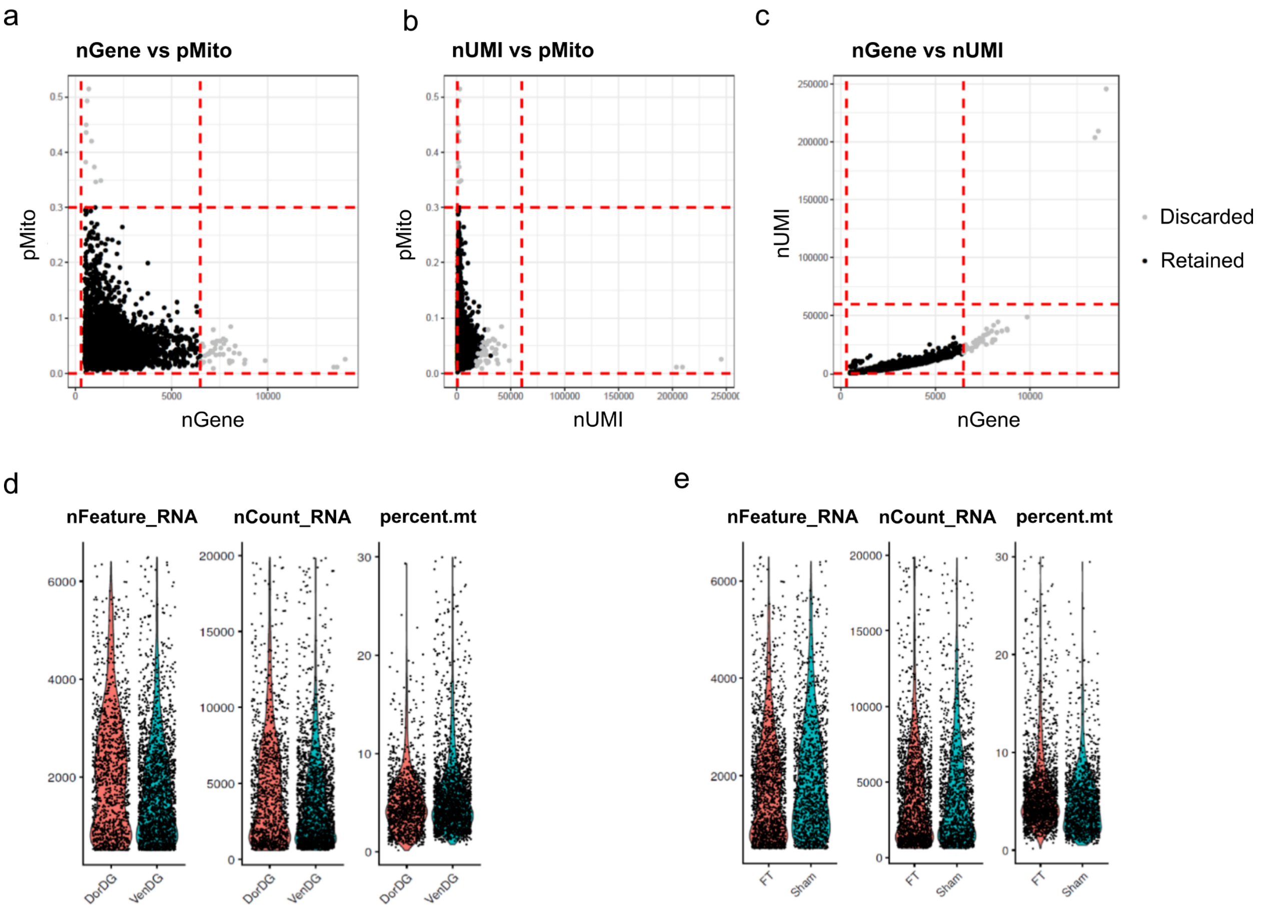

Supplementary Fig. S8

- **a.** Quality control for single-cell RNA-seq. All single-cell data points were used to plot the number of detected genes (nGene) in each cell vs the per-cell fraction of mitochondrial reads mapped (pMito). Black dots indicate cells that passed the QC thresholds, grey dots indicate cells were discarded from further downstream analysis.
- **b.** Number of unique molecular tags detected per cell (nUMI) vs pMito.
- **c.** Plot of nGene vs nUMI.
- **d.** nGene, nUMI and pMito distribution of all high-quality cells in dorDG and venDG single-cell datasets are shown.
- **e.** nGene, nUMI and pMito distribution of all high-quality cells in FT and Sham groups are shown.

Supplementary Fig. S8

f

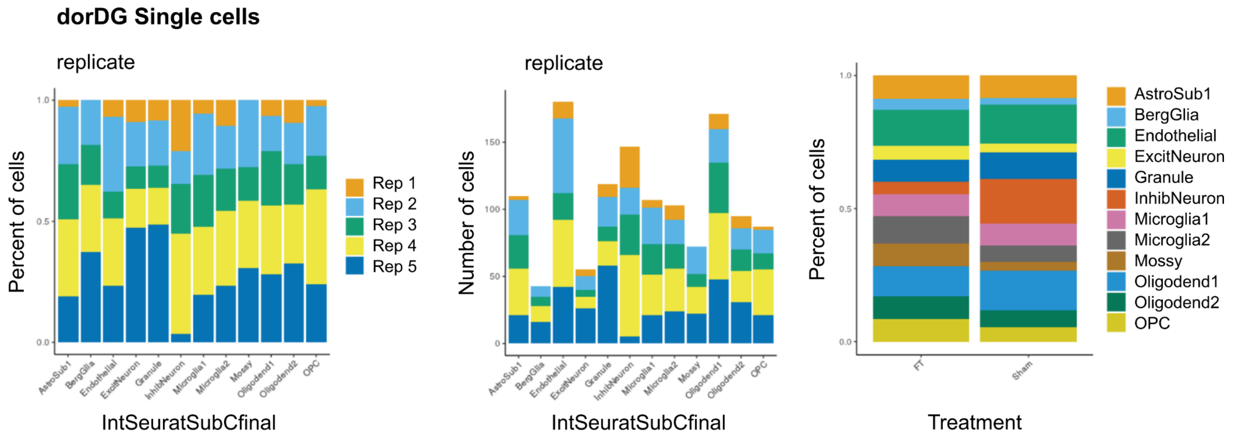

g

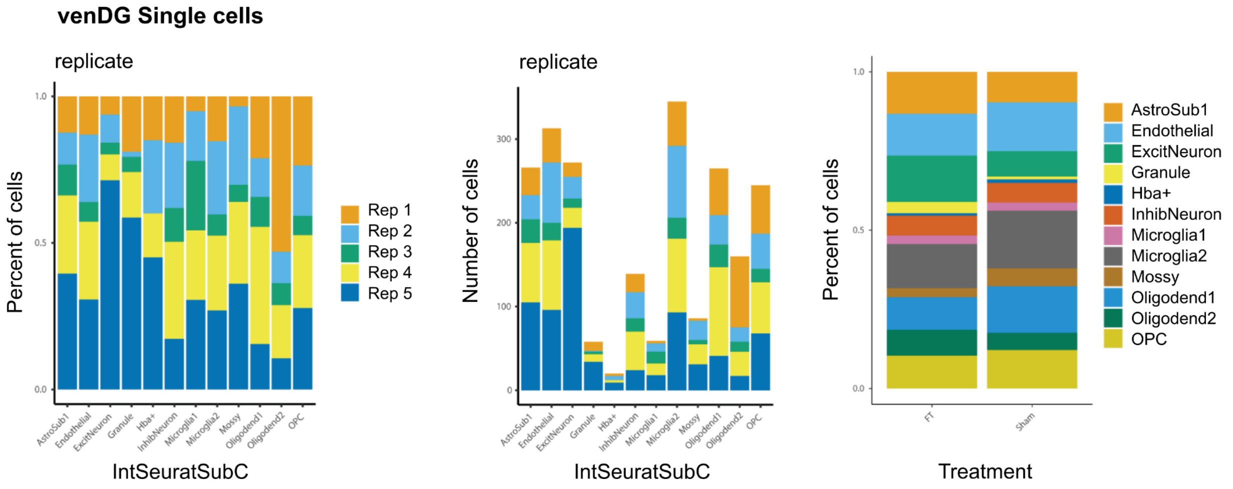

Supplementary Fig. S8 (continued)

f. For dorDG: *Left*, for each of the twelve annotated cell-types (x-axis), the fraction of cells contributed from each biological replicate; *middle*, for each of the twelve annotated cell-types (x-axis), the actual number of cells from each biological replicate; *right*, fractional proportions of the twelve annotated cell-types within FT and Sham.

g. As in **f.**, but for venDG.

Supplementary Fig. S9

a

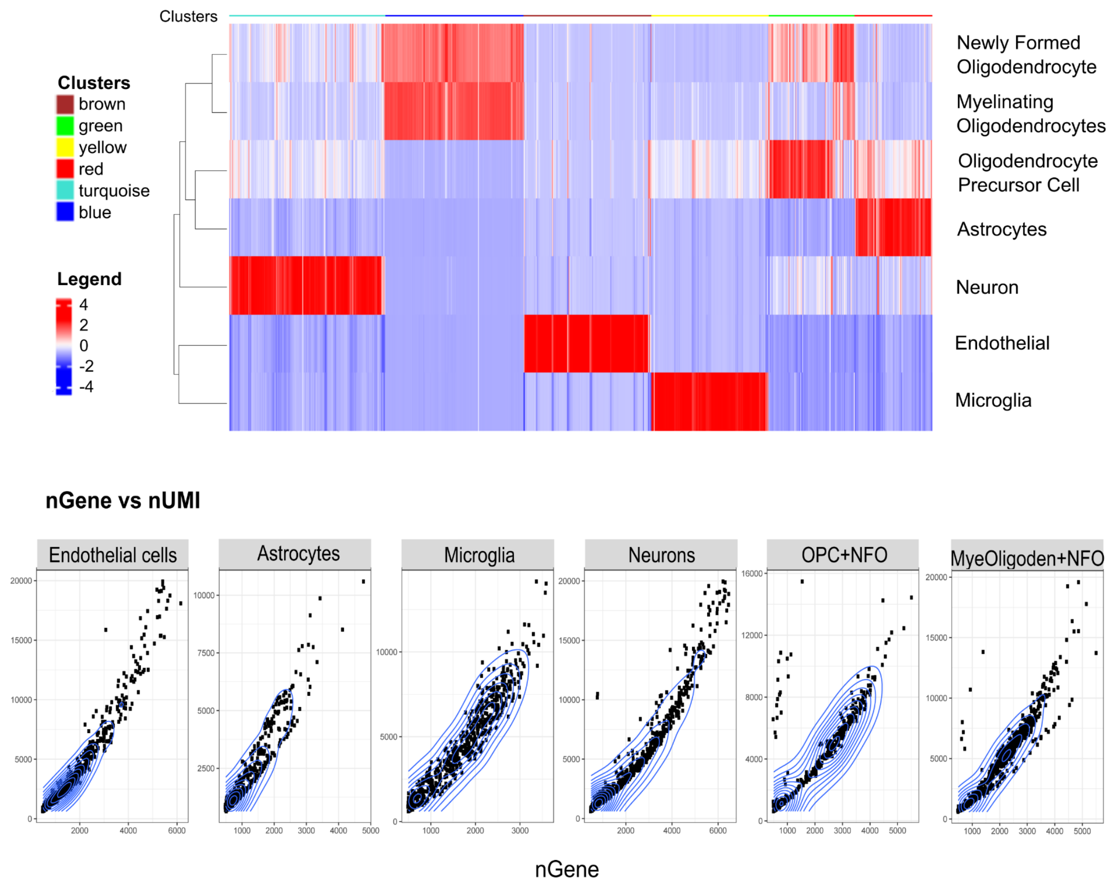

b

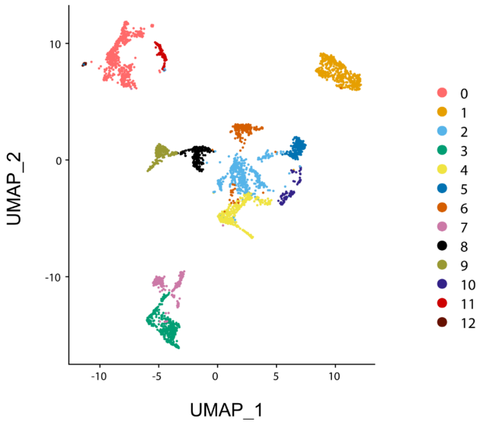

Supplementary Fig. S9

a. *Top:* supervised RCA2 clustering of the single-cells that passed QC (dorDG and venDG combined) were clustered into 7 major cell types using RCA2. *Bottom:* nGene vs nUMI for each cluster, with their corresponding density plots.

b. Unsupervised clustering results from Seurat, plotted as a UMAP: Following major cell type annotation and clustering using RCA2, each major cluster was subdivided into sub clusters. The UMAP shows 13 cell subtypes obtained by Seurat clustering (dims: 30, resolution: 0.8).

Supplementary Fig. S9

c

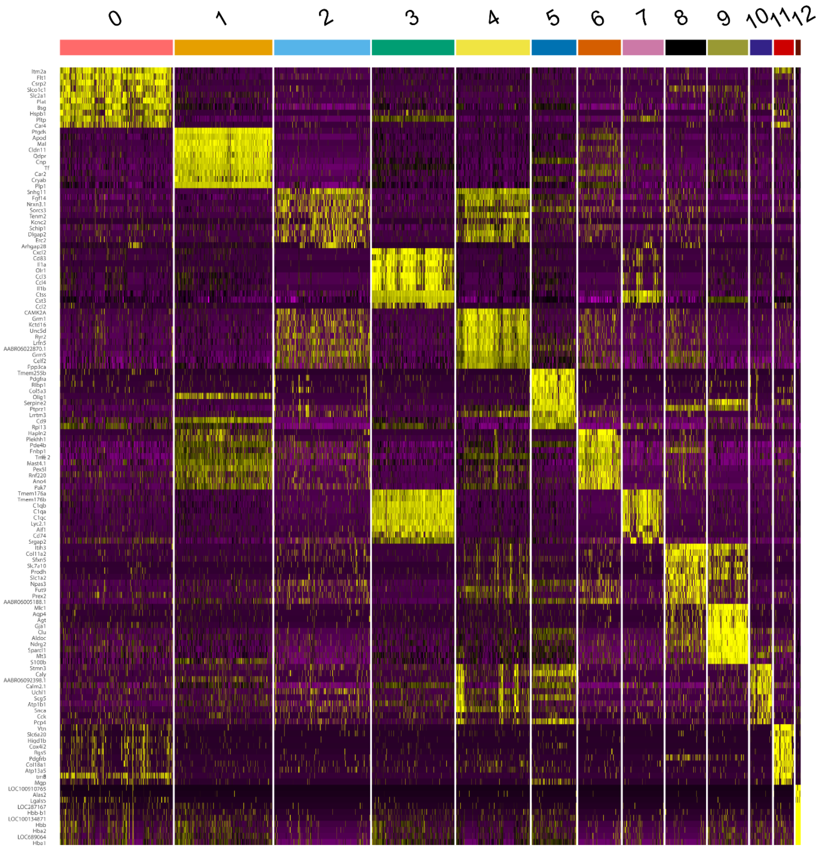

d

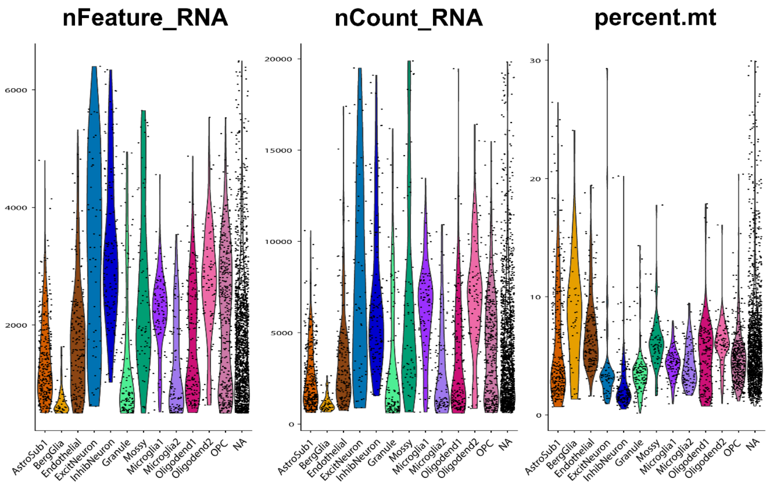

Supplementary Fig. S9 (continued)

- **c.** Heatmap of the log transformed gene expression values for the top 10 cell type specific markers. Single cells from dorDG and venDG (combined scRNA-seq dataset are shown here).
- **d.** nGene, nUMI and pMito distribution of each of the 13 annotated cell types. (Note: The Hba+ cluster in venDG was discarded from all downstream analysis.)

Supplementary Fig. S9

e

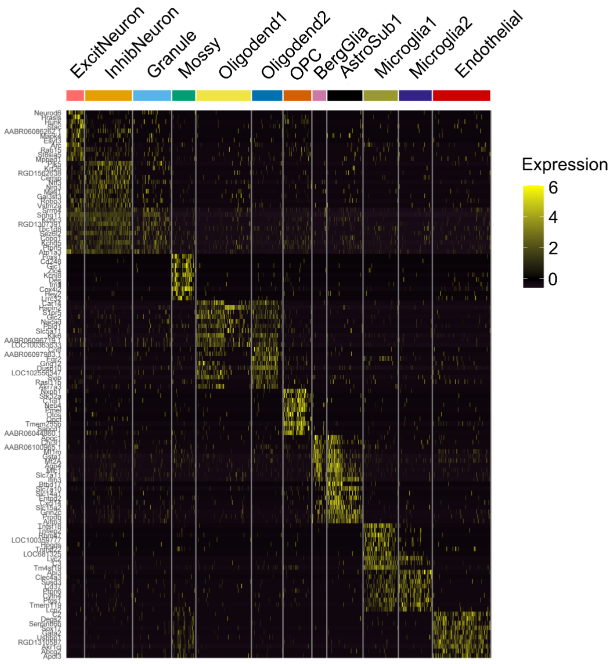

f

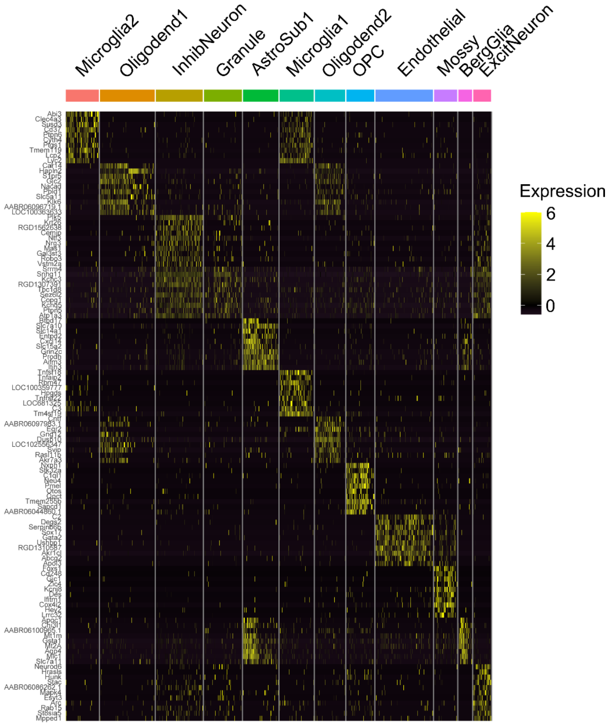

Supplementary Fig. S9 (continued)

- **e.** Heatmap showing the log-transformed gene expression values for the top 10 cell type specific markers identified in the dorDG sc-dataset.
- **f.** Heatmap showing the log-transformed gene expression values for the top 10 cell type specific markers identified in the venDG sc-dataset.

Supplementary Fig. S10a

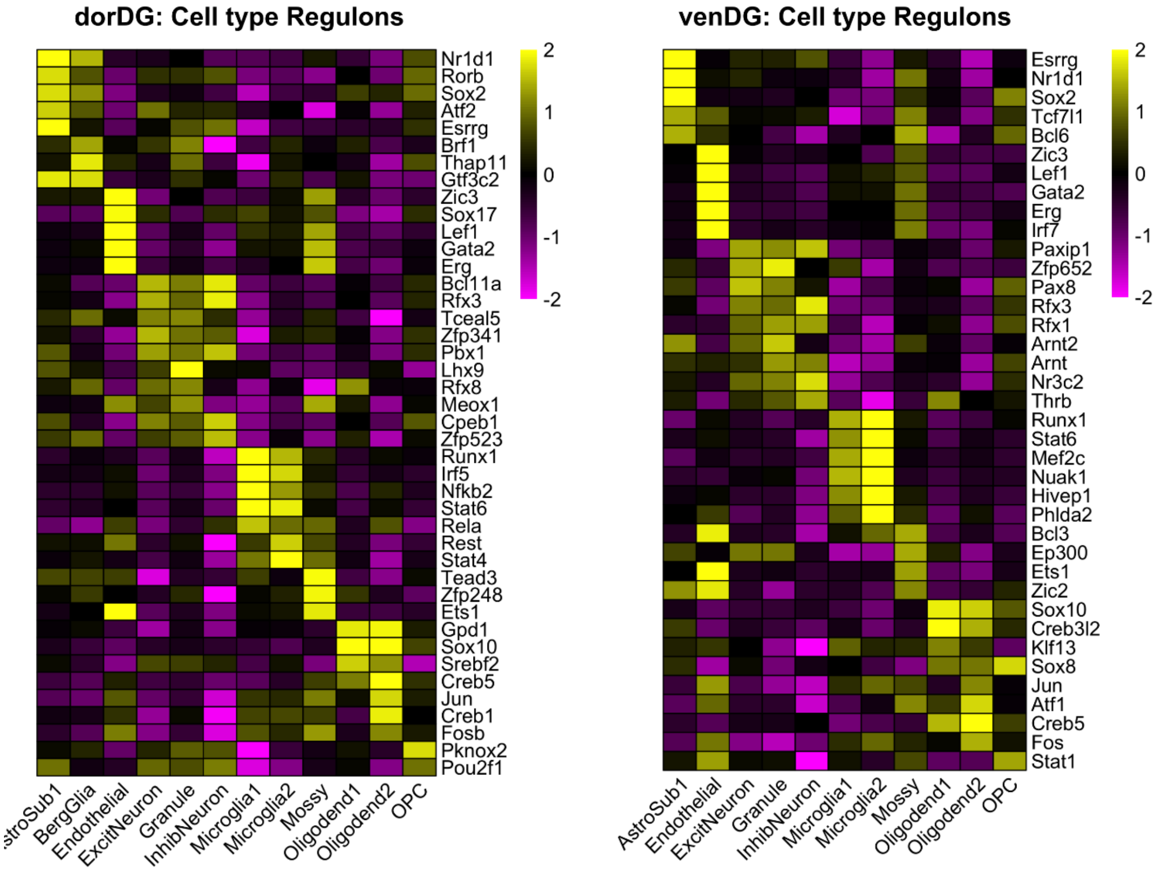

Supplementary Fig. S10

a. Heatmap of regulons identified in the different cell types within dorDG (left) and venDG (right). Heatmap is coloured by scaled regulon activity score (AUC Score), yellow denotes activated regulons and purple indicates inhibited regulons.

Supplementary Fig. S10b

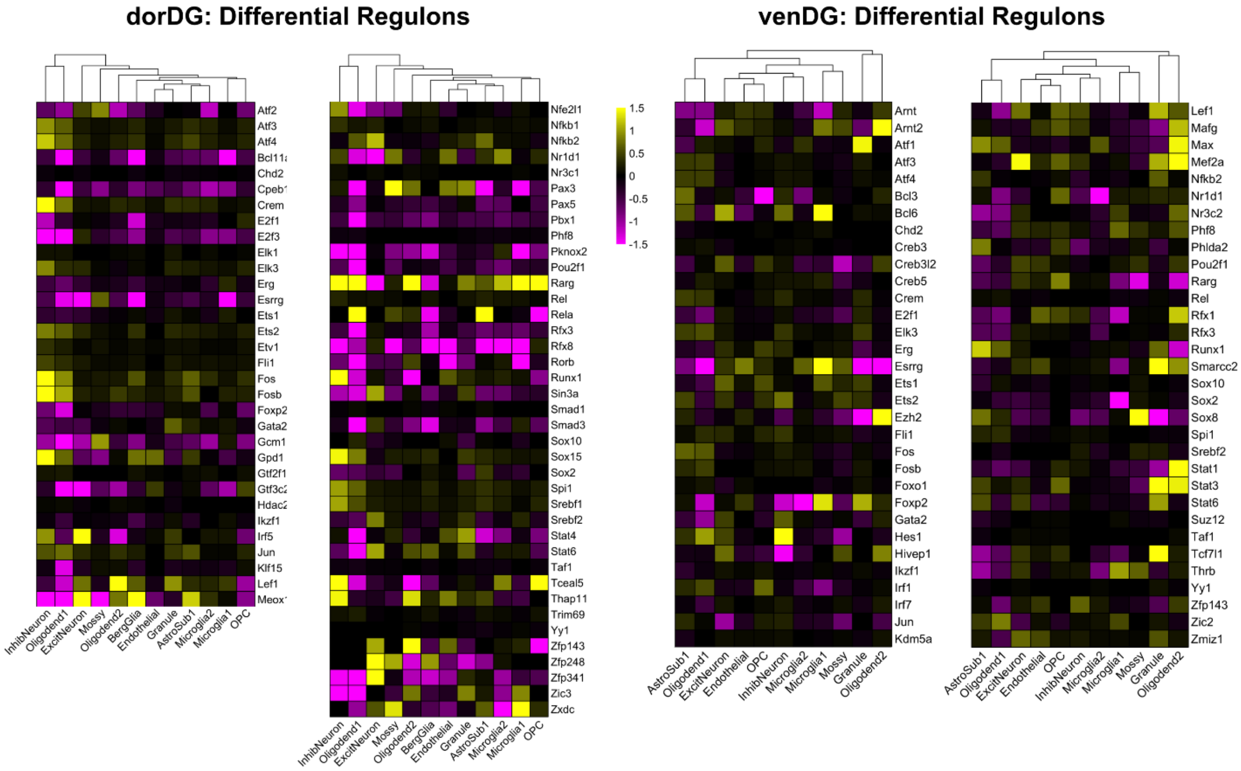

Supplementary Fig. S10

b. Heatmap of differentially activated regulons (FDR *Q-val* $\leq$0.2) identified in the different cell types within dorDG (left) and venDG (right). Heatmap is coloured by scaled regulon activity score (AUC Score), yellow denotes activated regulons and purple indicates inhibited regulons. For clarity, only regulons that are "non-extended" are shown.

Supplementary Fig. S11a

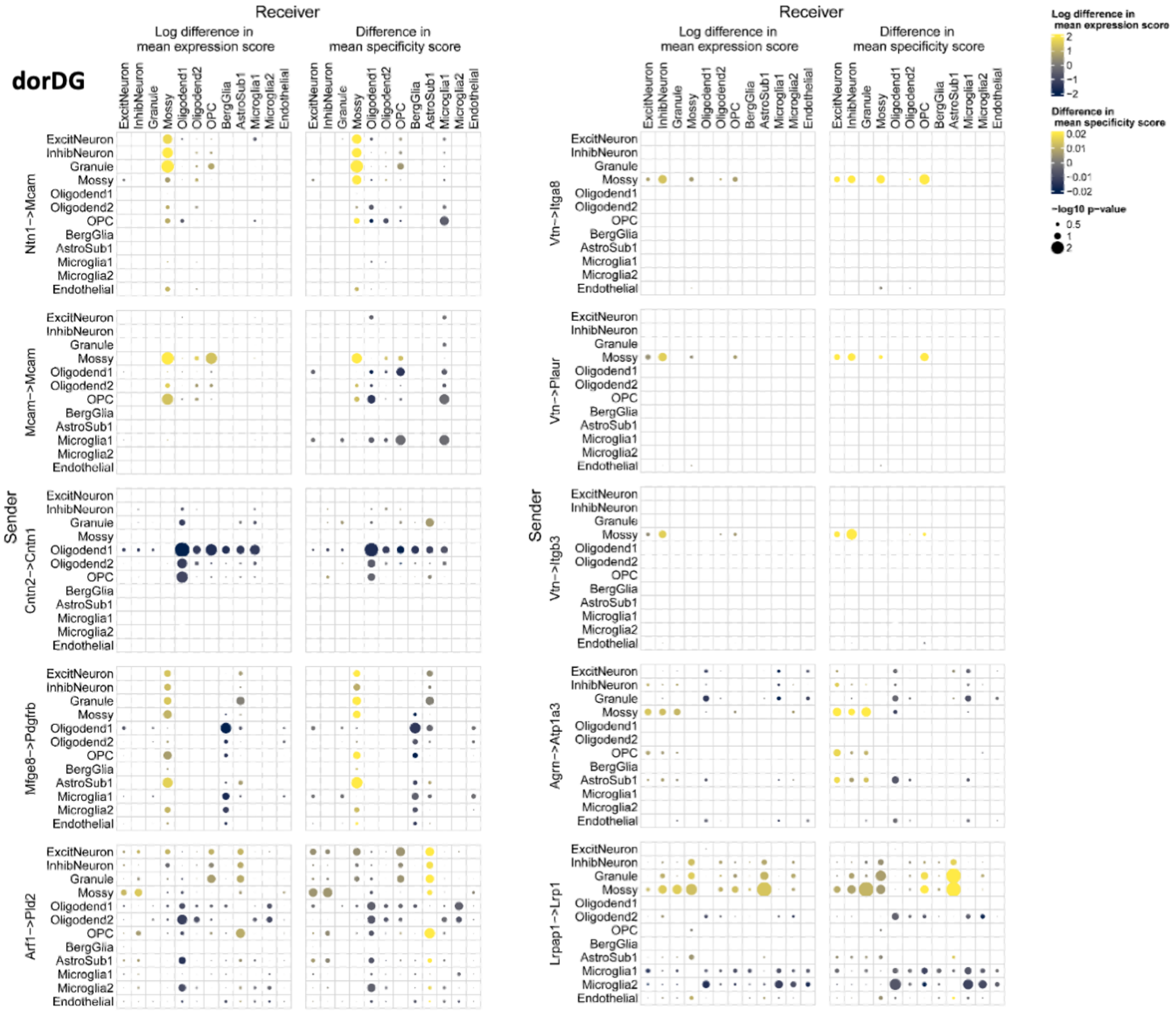

Supplementary Fig. S11

a. NATMI results (dorDG, continued on the next page): Log-fold change between average of NATMI expression scores (left column) and difference between average of NATMI specificity scores (right column) for top differential interactions between FT and Sham. More positive indicates a higher score in FT (blue), while more negative indicates a higher score in Sham (yellow). Dot size corresponds to Wilcoxon p-value testing for difference in expression or specificity score in FT. Only interactions where the ligand is expressed in at least 10% of cells in the sender cell type and the receptor is expressed in at least 10% of cells in the receiver cell type are depicted.

Supplementary Fig. S11a

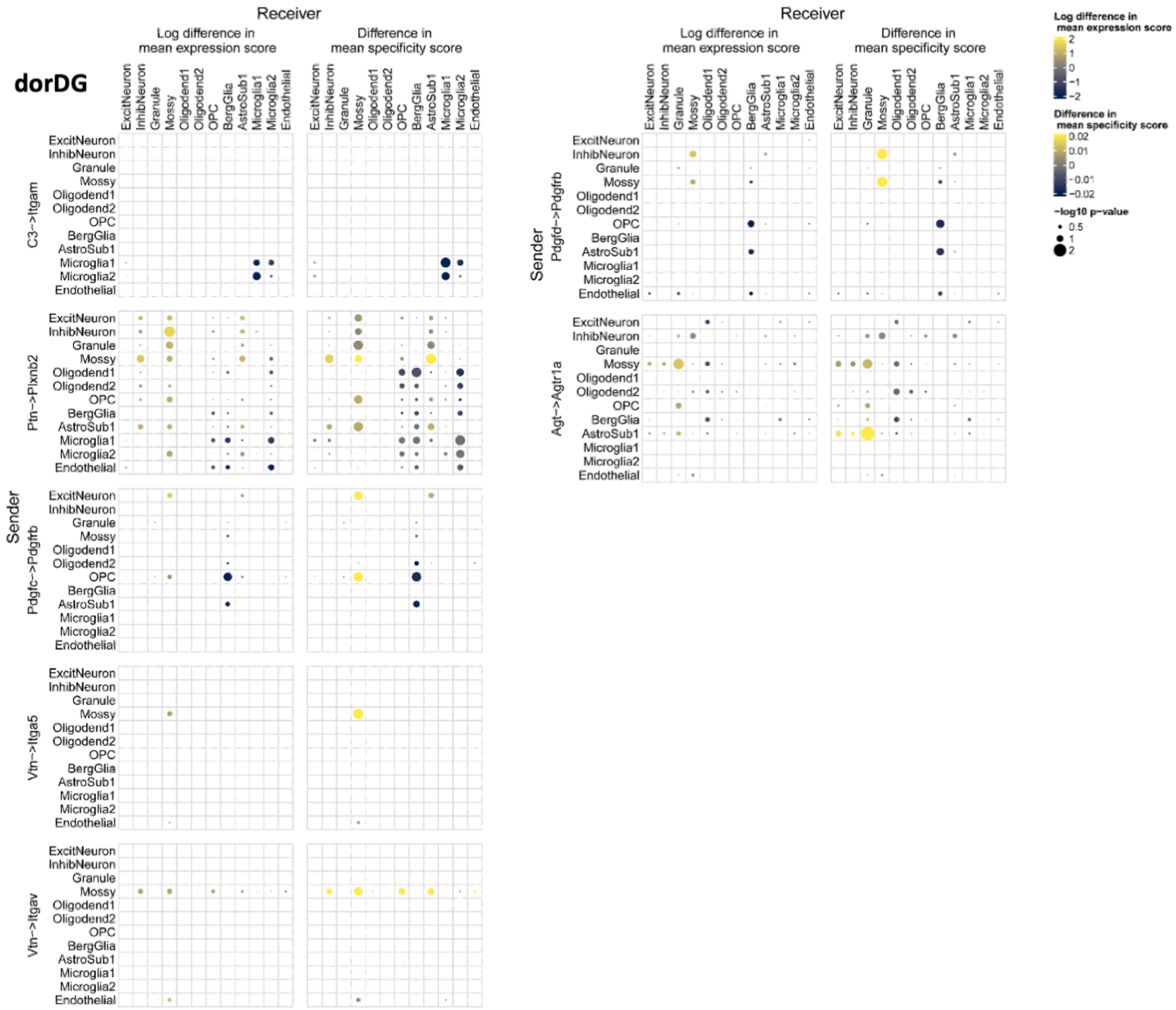

Supplementary Fig. S11a (continued)

Supplementary Fig. S11b

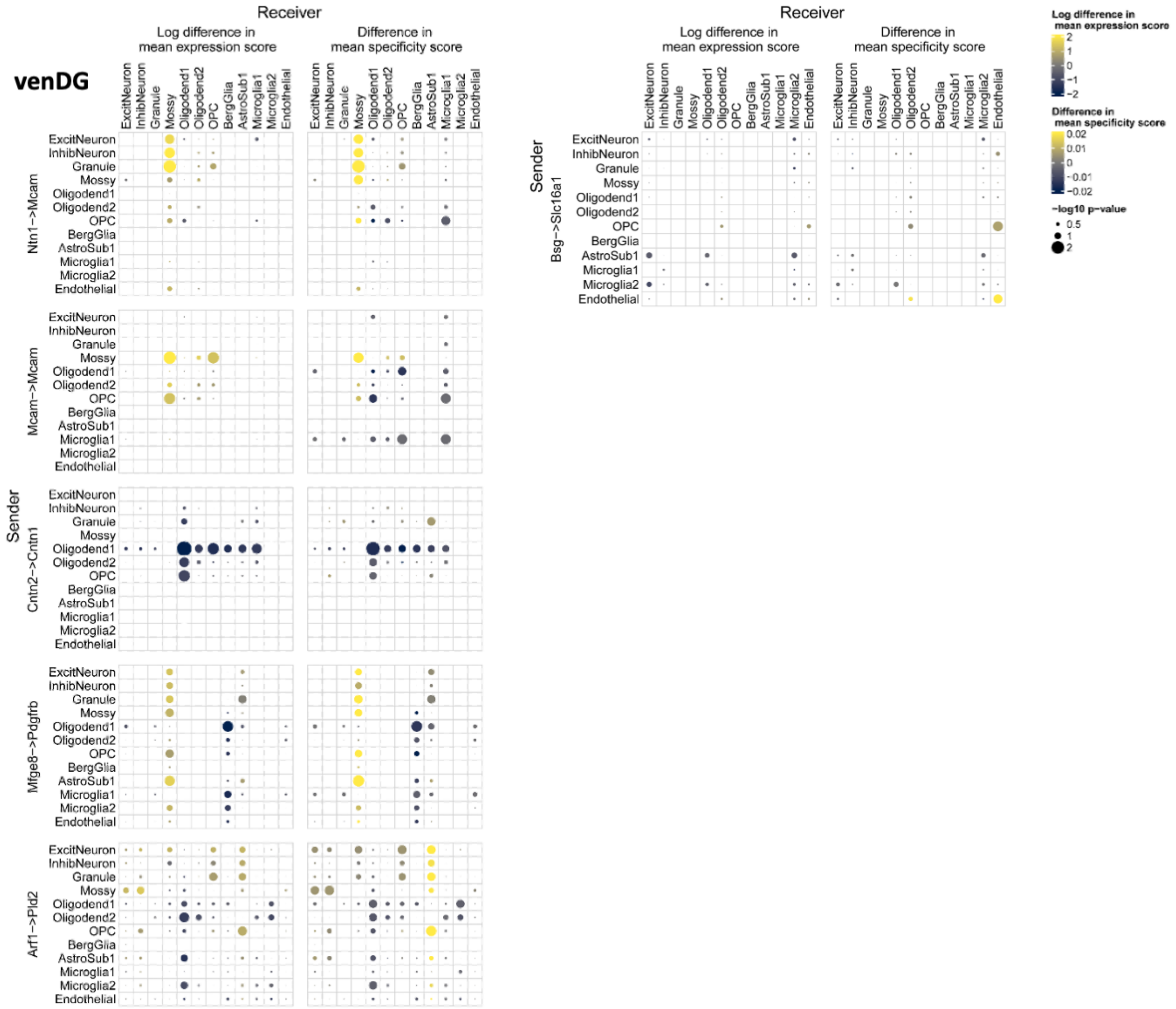

Supplementary Fig. S11b

b. NATMI results (venDG): Log-fold change between average of NATMI expression scores (left column) and difference between average of NATMI specificity scores (right column) for top differential interactions between FT and Sham. More positive indicates a higher score in FT (blue), while more negative indicates a higher score in Sham (yellow). Dot size corresponds to Wilcoxon p-value testing for difference in expression or specificity score in FT. Only interactions where the ligand is expressed in at least 10% of cells in the sender cell type and the receptor is expressed in at least 10% of cells in the receiver cell type are depicted.
